# Chemoautotrophic Thermodesulfobacteriota as a key genomic potential group in the hypoxic diazotrophic community of the Changjiang (Yangtze River) estuary

**DOI:** 10.3389/fmicb.2025.1671267

**Published:** 2025-12-04

**Authors:** Mengjia Zhang, Yuanli Zhu, Zhenhao Sun, Bin Wang, Jianfang Chen, Feng Zhou, Jiangning Zeng, Meng Li, Dayu Zou, Zhibing Jiang

**Affiliations:** 1 Key Laboratory of Marine Ecosystem Dynamics, Second Institute of Oceanography, Ministry of Natural Resources, Hangzhou, China; 2 Key Laboratory of Nearshore Engineering Environment and Ecological Security of Zhejiang Province, Hangzhou, China; 3 State Key Laboratory of Satellite Ocean Environment Dynamics, Second Institute of Oceanography, Ministry of Natural Resources, Hangzhou, China; 4 Observation and Research Station of Marine Ecosystem in the Yangtze River Delta, Ministry of Natural Resources, Hangzhou, China; 5 Archaeal Biology Centre, Synthetic Biology Research Center, Shenzhen Key Laboratory of Marine Microbiome Engineering, Key Laboratory of Marine Microbiome Engineering of Guangdong Higher Education Institutes, Institute for Advanced Study, Shenzhen University, Shenzhen, China; 6 Key Laboratory of Ocean Space Resource Management Technology, Ministry of Natural Resource, Marine Academy of Zhejiang Province, Hangzhou, China

**Keywords:** coastal hypoxia, nitrogen fixation, diazotrophic biodiversity, Thermodesulfobacteriota, metabarcoding and metagenomic analysis

## Abstract

Coastal hypoxia, intensified by global warming and eutrophication, profoundly affects marine nitrogen cycling. However, its impact on diazotrophic communities in large river estuaries remains poorly understood. During an unprecedented hypoxia event (minimum dissolved oxygen at 2.70 μmol L^−1^) in August 2016 in the Changjiang Estuary, we sampled across a dissolved oxygen (DO) gradient spanning hypoxic and non-hypoxic waters. Using *nifH* gene amplicon sequencing, metagenomic binning, and multivariate statistical analyses, we found that higher diazotrophic biodiversity was observed in hypoxia zone, with non-cyanobacterial diazotrophs dominating the communities. The phylum Thermodesulfobacteriota (with relative abundance of 58.93% totally) exhibited significant hypoxia-specific enrichment. LEfSe analysis identified Thermodesulfobacteriota as potential hypoxia biomarkers, while network analysis revealed their keystone role, representing 68.6% of highly connected nodes. Environmental drivers, including low DO concentrations (7.50–61.88 μmol L^−1^ in hypoxic vs. 66.56–255.63 μmol L^−1^ in non-hypoxic zones), elevated salinity (30.67–34.50), increased dissolved reactive phosphorus (0.39–1.26 μmol L^−1^), and nitrate depletion (0.30–22.50 μmol L^−1^), collectively created favorable conditions for the development of the observed diazotrophic community under hypoxia. Metagenomic analysis revealed a hypoxia-driven increase in *nifH* gene abundance, with *nifH*-carrying metagenome-assembled genomes affiliated with Thermodesulfobacteriota showing approximately a 4.7-fold higher relative abundance in hypoxic zone compared to non-hypoxic zone. Reconstruction of metabolic pathways from metagenome-assembled genomes (MAGs) further suggested their potential involvement in both nitrogen fixation and carbon–sulfur cycling. Amplicon and metagenomic datasets consistently demonstrated Thermodesulfobacteriota’s predominant in hypoxia. These findings redefine estuarine nitrogen flux models by highlighting hypoxia-driven taxonomic and functional shifts in diazotrophic communities, and provide a foundation for assessing the potential microbial resilience and ecosystem risks in expanding coastal hypoxic zones. Our study underscores the genomic potential of Thermodesulfobacteriota as key players in the nitrogen cycle under hypoxia, a hypothesis that warrants future validation through direct activity measurements.

## 1 Introduction

Nitrogen is an essential nutrient that limits primary productivity in vast oceanic regions. The availability of nitrogen, particularly in its bioavailable forms (e.g., ammonium, nitrate), is therefore a key regulator of marine ecosystem dynamics and carbon cycling (Capone et al., 2005; Karl et al., 2002). In the marine nitrogen cycle, biological nitrogen fixation performed by diazotrophs serves as a critical source of new nitrogen, counterbalancing nitrogen losses through processes like denitrification and anammox (Karl et al., 2002; Shi et al., 2021). Using the enzyme nitrogenase, diazotrophs convert dissolved molecular nitrogen (N_2_) into ammonia (NH₃), providing a vital external nitrogen source for the ocean (Karl et al., 2002; Zehr and Capone, 2024). Historically, this process is attributed to phototrophic cyanobacteria inhabiting oligotrophic surface waters (Gallon, 2001; Karl and Letelier, 2008). However, emerging evidence have revealed that diverse non-cyanobacterial diazotrophs (NCDs), including heterotrophic and chemoautotrophic bacteria and archaea, are widespread and contribute significantly to nitrogen fixation, especially in oxygen-deficient zones (Farnelid et al., 2011; Liu et al., 2012; Wu et al., 2019; Zehr and Capone, 2020). These environments, characterized by low oxygen, elevated phosphorus (P), and often enhanced bioavailable iron (Fe), can favor diazotrophy by reducing the oxygen inhibition of the nitrogenase enzyme and potentially coupling it to nitrogen loss processes like denitrification and anaerobic ammonium oxidation (Deutsch et al., 2007; Löscher et al., 2020). Consequently, the expanding low-oxygen waters, driven by climate change and anthropogenic activities, are predicted to become increasingly significant niches for marine nitrogen fixation (Stramma et al., 2008; Zhang et al., 2010; Zhou et al., 2022; Saxena et al., 2023; Liu et al., 2024).

The Changjiang (Yangtze River) Estuary (CE), forming a large eutrophic system in the East China Sea, receives massive anthropogenic nutrient loads, leading to severe coastal seasonal hypoxia (dissolved oxygen (DO) concentration < 62.5 μmol L^−1^) in its bottom waters during summer (Li et al., 2002; Dai et al., 2011). While previous studies have documented shifts in bulk bacterial communities associated with hypoxia development (Liu et al., 2012, 2013), the composition, diversity, and environmental drivers of the diazotrophic community, especially the role of NCDs, in this complex estuary remain poorly constrained. Furthermore, the large input of terrestrial organic matter from the Yangtze River (Gao et al., 2012; Bao et al., 2015) creates a unique environment where both heterotrophic and chemoautotrophic diazotrophic pathways could be important, presenting a compelling case study to explore their dynamics. However, the specific identity, metabolic potential, and ecological role of the dominant diazotrophs that underpin nitrogen fixation in the hypoxic zones of large river estuaries like the CE remain largely unknown.

In August 2016, an unprecedented hypoxia event (bottom-water DO as low as 2.70 μmol L^−1^) was recorded in the CE (Li et al., 2024). Capitalizing on this event, we sampled across hypoxic and non-hypoxic waters. By integrating *nifH* gene amplicon sequencing, metagenomic analysis, and multivariate statistics, we aimed to: (1) characterize the structural composition and diversity of diazotrophic communities in hypoxic versus non-hypoxic waters; (2) identify specific hypoxia-indicator taxa and keystone groups within the co-occurrence network; (3) elucidate the key environmental factors shaping diazotrophic assemblages under hypoxia. Our results will represent a significant step toward understanding diazotrophic community dynamics under hypoxic conditions in the CE, laying a strong foundation for future research on estuarine diazotrophs in the Changjiang Estuary and other coastal systems.

## 2 Materials and methods

### 2.1 Sample collection and environmental parameter analysis

**Figure 1:**
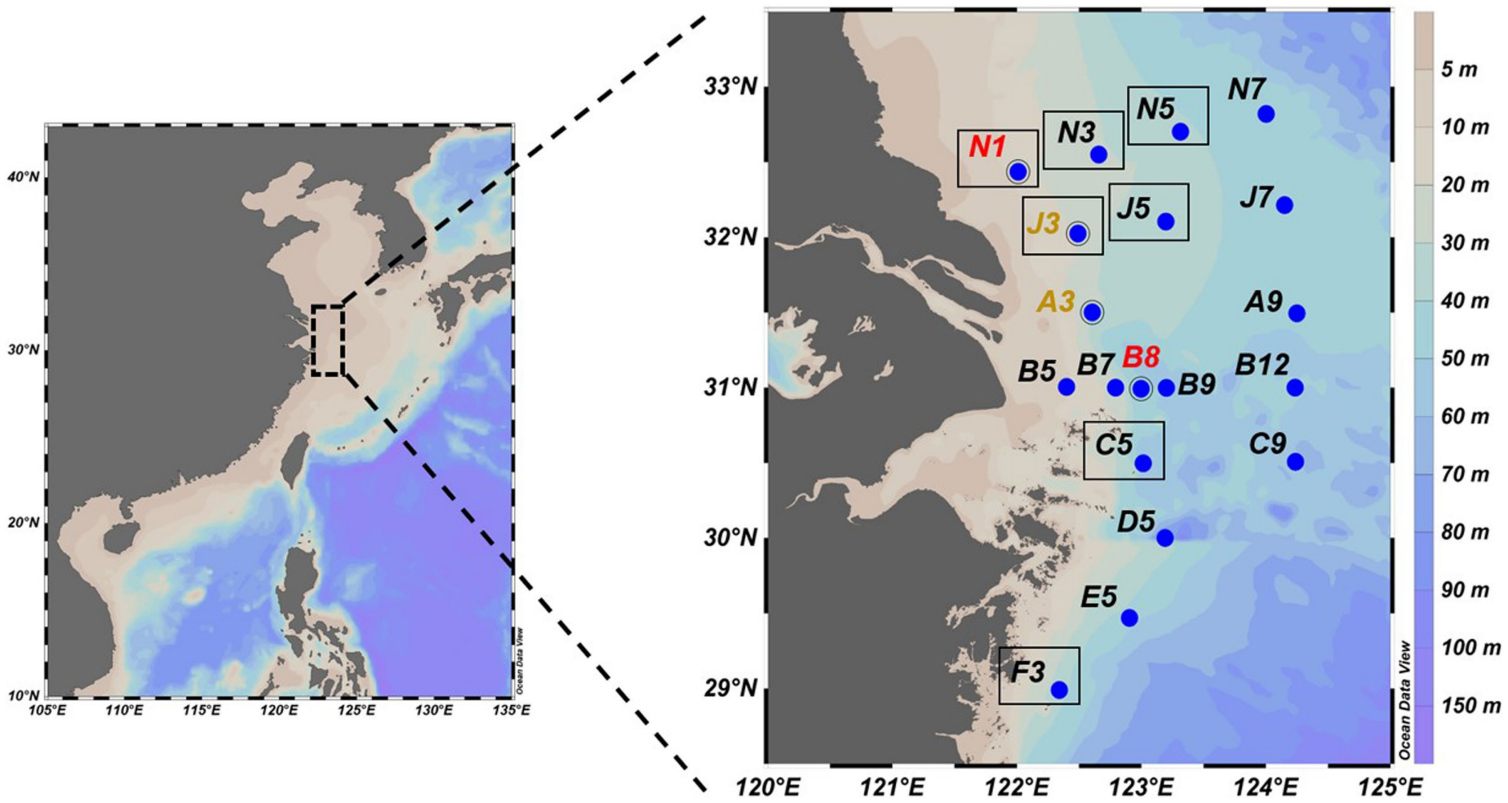
Spatial distribution of sampling stations in the Changjiang Estuary for this study. The inset map in the left shows the geographical location of the study area within China. Black labels represent stations with amplicon sequencing data; red labels indicate stations from Sun et al. (2024) containing metagenomic datasets, and the yellow label denotes stations with both data types. Black-bordered stations highlight those located within the hypoxic zone.

Water samples were collected from 19 stations in the CE during a research cruise (August 17–26, 2016) aboard R/V “*Run Jiang 1*” as part of the Long-term Observation and Research Plan in the Changjiang Estuary and the Adjacent East China Sea (LORCE) Project (Figure 1). This sampling design encompassed two complementary datasets. For *nifH* amplicon sequencing (metabarcoding), at each of the 17 stations, water samples were collected from both the surface (~ 2 m depth) and bottom (~ 2 m above the seabed) layers. This paired sampling design was implemented to capture the strong physicochemical gradient, particularly the DO stratification, characteristic of the estuarine system during summer. It allowed for a direct comparison between oxic surface and (potentially) hypoxic bottom waters within the same location, thereby controlling for horizontal spatial variability and strengthening the identification of hypoxia-associated microbial communities. Approximately 1 L of water from each depth was filtered sequentially through 0.2 μm polycarbonate membranes (Millipore, USA). Filtration commenced immediately upon retrieval of the Niskin bottles. The time from water collection to the completion of filtration was typically less than 30 min for each sample. Throughout this short period, samples were maintained indoor at ambient temperature to preserve their in-situ state until fixation. The filters were immediately flash-frozen in liquid nitrogen and stored at −80 °C until DNA extraction. For metagenomic sequencing, a subset of four stations was selected to represent key environmental gradients (two hypoxic and two non-hypoxic; Figure 1). This subset included two stations (J3, A3) that were shared with the amplicon dataset, and two additional stations (N1, B8) that were exclusively used for metagenomics. Metagenomic sequencing was performed on bottom-water samples from these four stations. The detailed procedures for the metagenomic sample collection are described in Sun et al. (2024). The station map was visualized using Ocean Data View v5.6.5 (Schlitzer, 2018) and clearly distinguished between stations used for amplicon, metagenomic, or both analyses.

Temperature, salinity, and water depth were recorded *in situ* at each site using a conductivity-temperature-depth (CTD) recorder (Sea-Bird Electronics, USA). DO concentrations were measured immediately after collection with the conventional Winkler titration method (Grasshoff and Ehrhardt, 1999) using the Mettler Toledo T50 potentiometric titrator. The sodium thiosulfate (Na₂S₂O₃) titrant was prepared daily at a low concentration (0.0020 N) to increase the titration volume for low-DO samples, thereby enhancing measurement resolution. This titrant was standardized daily against a certified potassium iodate (KIO₃) primary standard to ensure absolute accuracy. Based on repeated titrations of samples and blanks, the precision of our method was consistently better than ±0.5 μmol L^−1^. The method detection limit (MDL), calculated as 3 times the standard deviation of the procedural blank, was determined to be ~1.0 μmol L^−1^. Nutrients (nitrate, NO_3_^−^; dissolved reactive phosphorus, DRP) were analyzed later in the laboratory using a San^++^ continuous flow autoanalyzer (Skalar, Netherlands). The complete physicochemical dataset is publicly available on Figshare (doi: 10.6084/m9.figshare.28877102).

### 2.2 DNA extraction, PCR amplification and sequencing

For metabarcoding sequencing, total genomic DNA was extracted from the frozen filters using the HP Plant DNA Kit (Omega, USA) according to the manufacturer’s protocol. This kit was selected to optimize the lysis of a broad spectrum of diazotrophs, including cyanobacteria and NCDs. To ensure efficient disruption of all cell types, the standard lysis step was augmented by an additional 15-min incubation at 65 °C and vigorous vortexing for 1 min. The purity and quantity of the extracted DNA were assessed using a NanoDrop 2000c spectrophotometer (Thermo Fisher Scientific, USA). Based on stringent quality control criteria (A260/A280 ratio of 1.8–2.0 and concentration > 5 ng/μL), 23 high-quality DNA samples were obtained and processed for further analysis. The excluded sample did not meet these standards and was omitted to ensure the reliability of the sequencing data.

A nested polymerase chain reaction (PCR) strategy was employed for *nifH* gene amplification of the 23 samples using Q5® High-Fidelity DNA Polymerase (New England Biolabs, USA) in an MJ100 thermal cycler (Bio-Rad, USA). The first amplification was conducted using primers nifH4 (5’-TTYTAYGGNAARGGNGG-3′) and nifH3 (5’-ATRTTRTTNGCNGCRTA-3′) (Zehr and Turner, 2001) under the following conditions: initial denaturation at 95 °C for 5 min; 38 cycles of 94 °C for 1 min (denaturation), 52 °C for 1 min (annealing), and 72 °C for 1 min (extension); followed by a final extension at 72 °C for 7 min (Zehr and Turner, 2001). The Second amplification was performed using 1 μL of the primary product with primers nifH1 (5’-TGYGAYCCNAARGCNGA-3′) and nifH2 (5’-ADNGCCATCATYTCNCC-3′) (Zehr and Turner, 2001), under modified conditions of 40 cycles with a 59 °C annealing temperature (Zehr and Mcreynolds, 1989). PCR products were visualized on 2% agarose gel, and bands of the expected size (~ 360 bp) were excised and purified using the AxyPrep DNA Gel Extraction Kit (Axygen, USA). The purified amplicons were quantified, normalized, and constructed into libraries using the TruSeq Nano DNA LT Library Prep Kit (Illumina, USA). Paired-end sequencing (2 × 300 bp) was performed on an Illumina MiSeq platform by Personalbio Co., Ltd. (Shanghai, China). Raw sequence data have been deposited in the National Center for Biotechnology Information (NCBI) SRA under BioProject number PRJNA1183438.

For metagenomic sequencing, a total of four samples were selected for this analysis, provided by our collaborator. Detailed procedures for DNA extraction, metagenomic sequencing and assembly, and gene annotation are described in Sun et al. (2024).

### 2.3 Bioinformatic and statistical analysis

#### 2.3.1 Amplicon sequence processing

Raw amplicon sequences were processed using a Sliding Window Approach (window size = 10 bp, slide = 1 bp) with a 99% average accuracy threshold. Paired-end reads were merged using FLASH v1.2.7 (Magoc and Salzberg, 2011), requiring an overlap of > 10 bp with no mismatches. QIIME v1.8.0 (Caporaso et al., 2010) was used to demultiplex and filter sequences, removing: (1) Sequences < 150 bp; (2) Ambiguous bases (N); (3) Sequences with > 1 primer mismatch; (4) Sequences containing > 8 consecutive identical bases. Chimeras were removed using USEARCH v5.2.236, yielding high-quality sequences for Operational Taxonomic Unit (OTU) clustering via UCLUST (Edgar, 2010) at 97% similarity (Blaxter et al., 2005). The most abundant sequence within each OTU was selected as the representative sequence, which were taxonomically classified using BLASTn v2.10.0 + against the NCBI non-redundant nucleotide (nt) database (local, download in August 2024) and *nifH* ASV databases (Morando et al., 2024) under stringent parameters (e-value = 1e-5, qcov = 95%, identity = 99%). The OTU table was then rarefied to the minimum sequencing depth using the rrarefy function in R package VEGAN v2.6.6.1 (Dixon, 2003). The final dataset was then filtered using two sequential criteria to ensure high quality and biological relevance: (1) Abundance Filter: OTUs with a mean relative abundance below 0.001% of the total dataset were removed (Bokulich et al., 2013); (2) Taxonomic Filter: OTUs that could be confidently annotated at the genus level or below were retained for downstream ecological analysis.

#### 2.3.2 Metagenomic assembly, binning, and annotation

Raw metagenomic reads from the four samples were quality-trimmed using Fastp v0.23.2^1^ with default parameters. *De novo* co-assembly of all quality-filtered reads was performed using MEGAHIT v1.2.9^2^ with the meta-large preset. Contigs longer than 1,500 bp were binned into metagenome-assembled genomes (MAGs) using the binning module of MetaWRAP v1.3.2 (Uritskiy et al., 2018). Bin refinement was performed to obtain a high-quality set of MAGs. MAG quality (completeness and contamination) was assessed using CheckM v1.2.1 (Parks et al., 2015). Only MAGs that met the medium-quality threshold (≥ 50% completeness and < 10% contamination) as defined by the Minimum Information about a Metagenome-Assembled Genome (MIMAG) standard (Bowers et al., 2017) were retained for downstream taxonomic and functional analysis. MAGs were then taxonomically classified using GTDB-Tk v2.4.0 (Chaumeil et al., 2022) against the Genome Taxonomy Database (GTDB r207). The relative abundance of MAGs across samples was quantified by mapping reads back to the assembly using BBMap v38.87^3^ and calculating coverage with CoverM v0.6.1 (Samuel et al., 2024) using the ‘genome’ method in Reads Per Kilobase per Million mapped reads (RPKM). Functional gene annotation was performed against the Kyoto Encyclopedia of Genes and Genomes (KEGG) database using diting v2.0 (Xue et al., 2021) with an e-value cutoff of 1e-5. The metabolic potential of MAGs, particularly for nitrogen, carbon, and sulfur cycling, was profiled using METABOLIC v4.0 (Zhou et al., 2019).

#### 2.3.3 Statistical analyses

Rarefaction curve and accumulation analyses were first performed to ensure the sufficient sample size and sequencing depth using R package ggplot2 v3.4.2 (Villanueva and Chen, 2019). To clarify influence of DO concentrations on diazotrophic diversity and spatial distribution, amplicon samples were divided into four regions: hypoxic zone (all in the bottom, abbreviated as Hypoxic-Bottom, HB, DO < 62.5 μmol L^−1^, including seven samples J3-10S, J3-21S, N3-29S, J5-34S, N5-32S, F3-31S, and C5-58S), surface layers above the hypoxic zone without hypoxia (Hypoxic-Surface, HS, including three samples F3-02S, J3-02S, and J5-02S), bottom layers in the non-hypoxic zone (Non-hypoxic-Bottom, NB, including nine samples D5-57S, B7-20S, A9-20S, C9-50S, B12-20S, N7-40S, J7-37S, B9-20S, and E5-56S), and surface layers in the non-hypoxic zone (Non-hypoxic-Surface, NS, including four samples B5-02S, A3-02S, B9-02S, and J7-02S). Inter-region differences in environmental parameters were assessed using the Non-parametric Kruskal-Wallis (K-W) test in R software v3.6.1, with statistical significance set at *p* < 0.05. *Post hoc* pairwise comparisons were conducted using Nemenyi’s test with Benjamini-Hochberg adjustment for multiple comparisons. Alpha and beta diversity metrics were calculated based on the rarefied OTU table. Alpha diversity index was calculated using the R package VEGAN, including Chao1, Shannon diversity, Simpson diversity, Abundance-based Coverage Estimator (ACE), Pielou’s evenness, and Good’s coverage. Beta diversity was assessed based on Bray-Curtis dissimilarities and visualized via principal coordinates analysis (PCoA) using the VEGAN package in R. To quantify the individual and combined contributions of environmental variables to the community composition, a permutational multivariate analysis of variance (PERMANOVA, ADONIS) test was performed using the adonis2 function in the R package VEGAN, with 999 permutations. The model included DO, temperature, salinity, NO_3_^−^, and DRP as explanatory variables. To further disentangle the correlation between community composition and environmental factors while controlling for the influence of co-varying variables, a Mantel test was performed using the mantel.partial function in the R package VEGAN. Besides, all pie charts, bar charts, and line charts were plotted using the R package ggplot2 v3.4.2 (Villanueva and Chen, 2019).

Linear discriminant analysis Effect Size (LEfSe v1.0) analysis (Segata et al., 2011) and random forest classifiers (Liaw and Wiener, 2002) were used to identify significant indicator groups. Specifically, LEfSe analysis employed the all-against-all strategy with linear discriminant analysis (LDA) score thresholds ≥ 3 on the Galaxy online analytics platform.^4^ Random forest analysis was conducted using the R package randomForest. Co-occurrence networks (Lu et al., 2018) were constructed to explore interactions within the diazotrophic community. OTUs with prevalence ratio > 20% and total relative abundance > 0.01% were selected for co-occurrence network construction based on strong (|r| > 0.7) and significant (*p* < 0.01) Spearman correlations (r) using the igraph package. Keystone groups were identified based on their topological roles within the co-occurrence network. For the OTU-OTU network, the criteria for selecting key OTUs varied slightly in different samples. In this study, nodes that ranked within the top 20% for both degree and closeness centrality, while simultaneously ranking within the bottom 20% for betweenness centrality, were classified as keystone taxa (Guimera and Nunes Amaral, 2005; Banerjee et al., 2018; Xie et al., 2023). Consequently, keystone groups were defined as those with high degree (≥ 10.6), high closeness centrality (> 0.2), and low betweenness centrality (< 10). For identified indicator groups and network keystone taxa, correlations between them and environmental factors were calculated using Spearman’s method in the R package psych v2.1.9 and visualized with pheatmap v1.0.12. Phylogenetic reconstruction was performed using IQ-TREE v1.6.12 (Jana et al., 2016) under the maximum likelihood (ML) framework.

## 3 Results

### 3.1 Richness and relative abundance of diazotrophs

**Figure 2:**
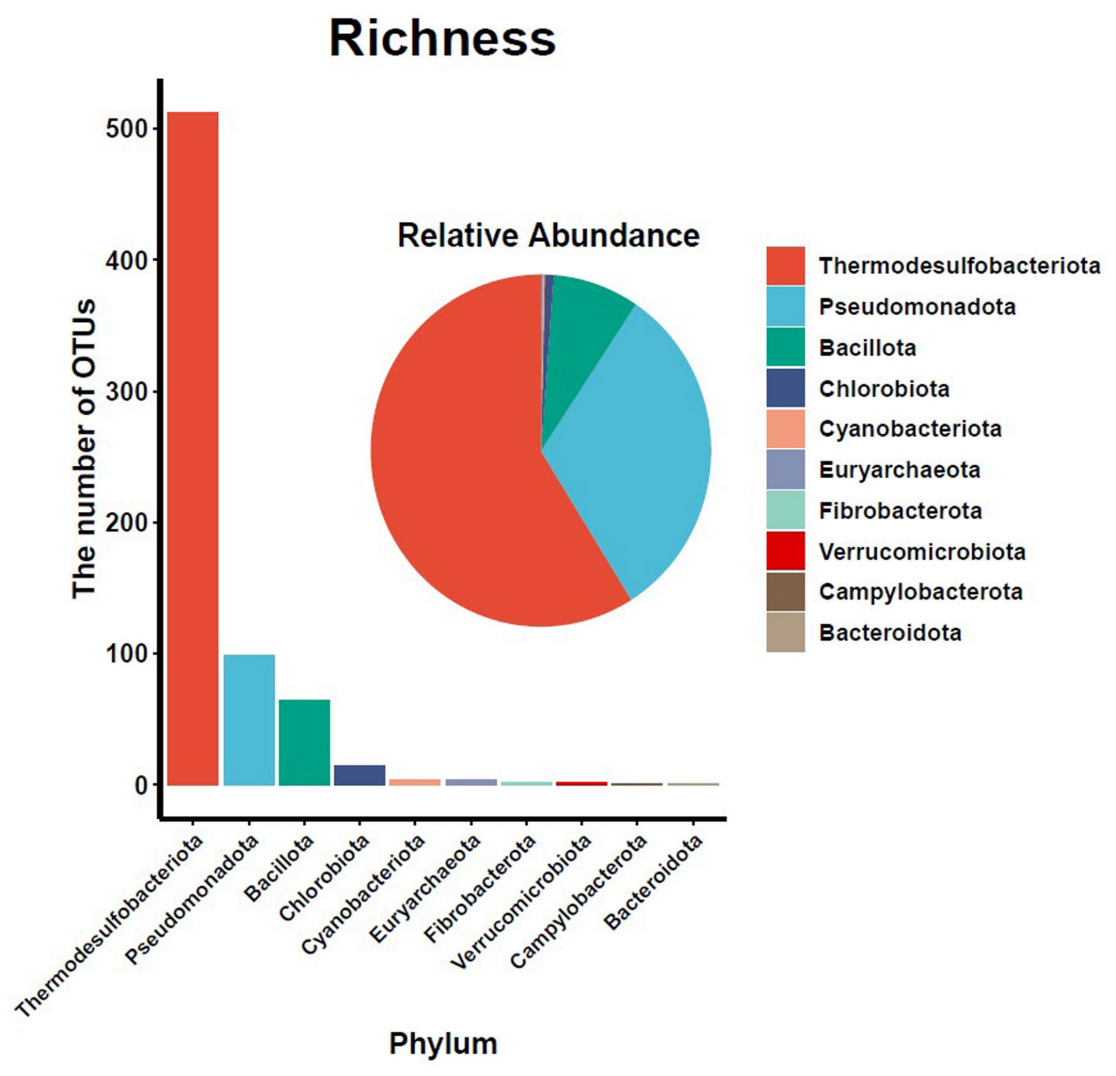
Phylogenetic composition of diazotrophic communities (from amplicon data) in the Changjiang Estuary. Bar heights represent taxonomic richness (number of OTUs), while pie chart areas reflect relative abundance of diazotrophs across bacterial and archaeal phyla.

A total of 12,602 OTUs were generated from *nifH* gene sequencing of 23 water samples, providing high taxonomic resolution at the genus/species level (Supplementary Figure S1). After filtering mentioned in 2.3.1, 757 OTUs were retained for downstream analyses. Among these, four archaeal OTUs were assigned to the phylum Euryarchaeota, while the remaining 753 bacterial OTUs were distributed across nine phyla (Figure 2). The diazotrophic community exhibited pronounced phylogenetic dominance, with Thermodesulfobacteriota accounting for 58.93% of total relative abundance (512 OTUs), followed by Pseudomonadota at 31.67% (99 OTUs; Figure 2). Besides, typical nitrogen-fixing Cyanobacteriota were negligibly represented, comprising only 0.17% of total abundance across 4 OTUs (Figure 2), suggesting potential environmental constraints on their proliferation in this estuarine system.

### 3.2 Environmental factors and spatial differences

Physicochemical parameters varied significantly among the four regions, which were classified based on DO concentrations (Table 1). Non-parametric K-W test results indicated that DO concentrations differed significantly (*p* = 0.0004 < 0.001) between every two groups, except between HS and NS. Temperature and salinity also showed significant differences (*p* = 0.005 < 0.01 and *p* = 0.002 < 0.01, respectively), while it reflected in layers (surface and bottom) instead of stations. Nutrient analysis revealed that HB had notably lower NO_3_^−^ concentrations compared to other regions, with significant differences across all comparisons (*p* = 0.02 < 0.05). DRP concentrations were significantly lower in the HS compared to other groups (*p* = 0.02 < 0.05).

**Table 1:** Regional differences of environmental variables in four regions by using Kruskal–Wallis Test.

Environmental factors	HB (Range; Mean ± SD)	HS (Range; Mean ± SD)	NB (Range; Mean ± SD)	NS (Range; Mean ± SD)	Significant differences
DO (μmol L^−1^)	7.50–61.88; 32.77 ± 21.37 ^a^	206.56–227.50; 216.25 ± 10.56 ^b^	66.56–117.19; 93.60 ± 18.65 ^c^	89.69–255.63; 179.77 ± 68.26 ^b^	*P* = 0.0004 < 0.001
Temperature (°C)	19.36–23.20; 21.65 ± 1.38 ^a^	28.62–30.52; 29.77 ± 1.01 ^b^	16.98–29.37; 20.82 ± 3.66 ^a^	26.38–31.36; 28.69 ± 2.05 ^b^	*P* = 0.005 < 0.01
Salinity	30.67–34.50; 32.41 ± 1.31 ^a^	16.10–27.76; 21.32 ± 5.92 ^b^	31.13–34.55; 33.37 ± 1.33 ^a^	15.51–28.85; 21.16 ± 5.58 ^b^	*p* = 0.002 < 0.01
NO_3_^−^ (μmol L^−1^)	0.30–22.50; 10.07 ± 9.10 ^a^	2.41–22.73; 13.06 ± 10.20 ^b^	1.37–21.74; 13.93 ± 6.63 ^b^	12.87–49.89; 34.52 ± 16.48 ^c^	*p* = 0.02 < 0.05
DRP (μmol L^−1^)	0.39–1.26; 0.71 ± 0.34 ^a^	0.01–0.02; 0.02 ± 0.01 ^b^	0.05–1.05; 0.59 ± 0.32 ^a^	0.05–0.72; 0.29 ± 0.38 ^a^	*P* = 0.02 < 0.05

DO = dissolved oxygen (μmol L^−1^); DRP = dissolved reactive phosphorus (μmol L^−1^); HB = Hypoxic-Bottom, represents hypoxic zone (all in the bottom, DO < 62.5 μmol L^−1^); HS = Hypoxic-Surface, represents surface layers above the hypoxic zone without hypoxia; NB = Non-hypoxic-Bottom, represents bottom layers in the non-hypoxic zone; NS = Non-hypoxic-Surface, represents surface layers in the non-hypoxic zone; Different superscript letters indicate significant differences between groups (*p* < 0.05, Kruskal-Wallis test with Nemenyi’s post hoc adjustment).

The metagenomic subset included two hypoxic (J3, N1) and two non-hypoxic (A3, B8) bottom-water samples, strategically selected to capture redox transition zones. This sampling design enabled a comparative analysis of metabolic potential across oxygen gradients, which is elaborated in the subsequent functional sections.

### 3.3 Diazotrophic community composition associated with oxygen availability

**Figure 3:**
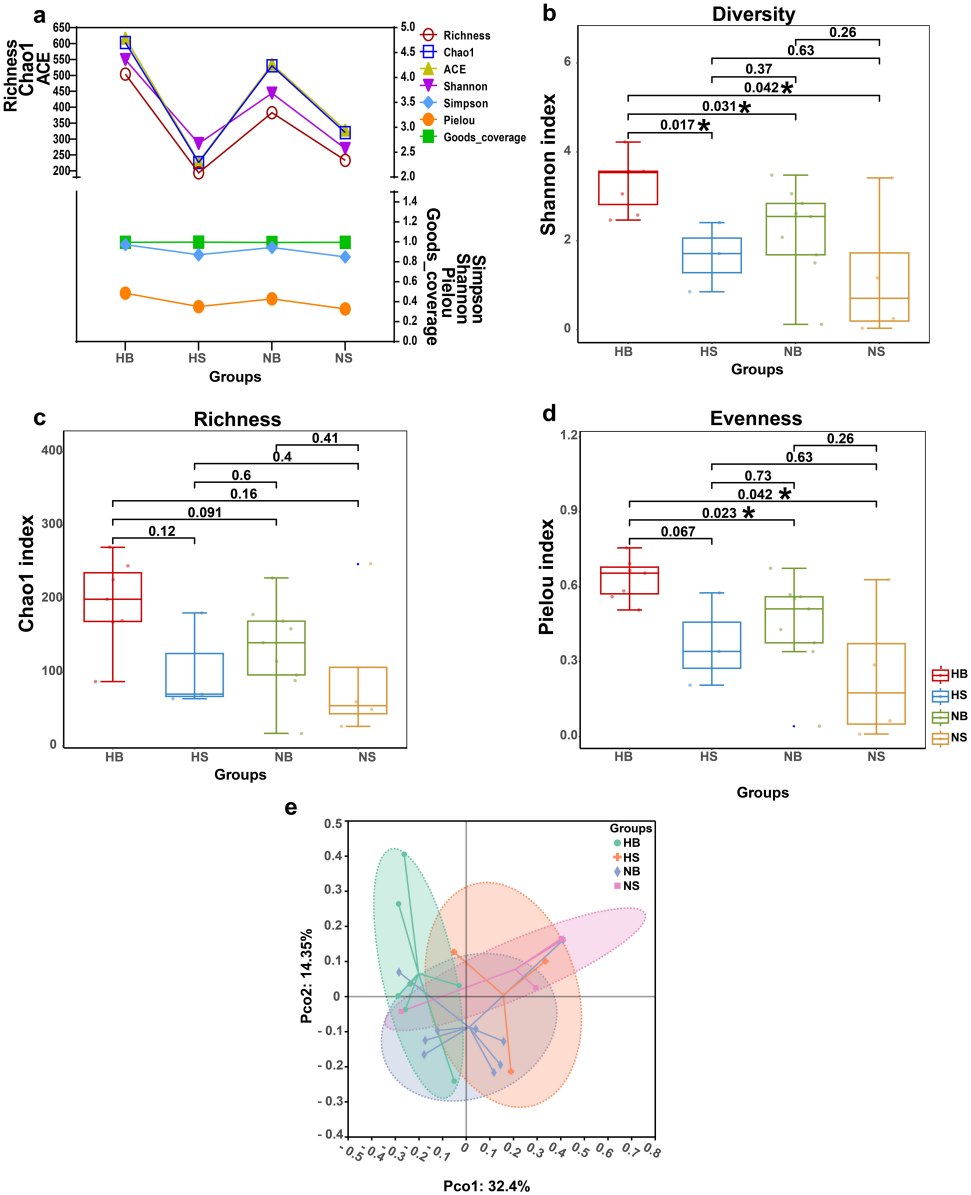
DO-dependent stratification of diazotrophic alpha and beta diversity index: **(a)** Composite metrics, **(b)** Shannon diversity (both the quantity and evenness of the species), **(c)** Phylogenetic richness (Chao1 index, quantity of species), **(d)** Community evenness (Pielou index), and **(e)** Principal coordinate analysis (PCoA) based on Bray-Curtis distances. Box plots display median values (central lines), interquartile ranges (boxes), and 1.5 × IQR whiskers; Asterisks (*) indicate significant intergroup differences (Kruskal-Wallis test, *p* < 0.05).

The composition of diazotrophic communities varied significantly along the DO gradient. Rarefaction curves reached asymptotes at ~10,000 sequences per sample, indicating sufficient sequencing depth. Species accumulation curves plateaued at 23 samples, confirming comprehensive community capture (Supplementary Figure S2). Alpha diversity was significantly higher in the HB region than elsewhere, with Shannon index values 1.2 times greater than in NB waters, 1.6 times greater than in HS waters, and 1.7 times greater than in NS waters (*p* = 0.017, 0.031, and 0.042, respectively; Figures 3a,b). In contrast, richness estimates (Chao1 index) showed no significant differences among regions (Figure 3c). Evenness, however, was markedly higher in both surface and bottom water of hypoxic sites (HB and HS) compared with non-hypoxic sites (NB and NS), as indicated by the Pielou index (*P* (HB-NB) = 0.023 < 0.05, *P* (HB-NS) = 0.042 < 0.05; Figure 3d).

Multivariate analysis revealed clear biogeographic structuring of diazotrophic communities. PCoA based on Bray–Curtis dissimilarity explained 46.75% of the total variance (PCo1: 32.4%, PCo2: 14.35%). Although separation between clusters was not absolute, samples from the HB region showed a distinct trend along PCo1. The overall community differentiation was statistically significant but exhibited a weak effect size (ANOSIM R = 0.169, *p* = 0.041; Figure 3e). To better quantify the influence of environmental factors, a PERMANOVA was conducted, which revealed that a model including DO, temperature, salinity, NO_3_^−^, and DRP explained 36.36% of the total community variance (Supplementary Table S1). Among these, DO was the most significant but not solitary driver, uniquely explaining 9.69% of the variance (*p* < 0.001), and other co-varying environmental parameters, including temperature (R^2^ = 8.44%, *p* < 0.001), salinity (R^2^ = 6.42%, *p* = 0.012) and NO_3_^−^ (R^2^ = 6.71%, *p* = 0.013) also collectively exert an influence on diazotrophic community composition. To further dissect the specific environmental associations of the dominant diazotrophic phyla, we performed Mantel tests between each phylum’s abundance and key environmental variables (Supplementary Figure S4). The analysis revealed that no single environmental factor exerted a strong (Mantel’s r > 0.4), universal control over the three major phyla. Instead, each phylum exhibited a distinct and relatively weak response pattern to the environmental gradients. The heterotrophic Pseudomonadota showed the strongest individual correlations, being significantly influenced by temperature (Mantel’s r = 0.2–0.4, *p* = 0.01–0.05) and moderately associated with salinity (Mantel’s r = 0.2–0.4, *p* > 0.05) and DO (Mantel’s r = 0.2–0.4, *p* > 0.05). The phototrophic Cyanobacteria were moderately correlated with DO (Mantel’s r = 0.2–0.4, *p* > 0.05), consistent with their oxygen-sensitive lifestyle. Notably, the dominant phylum under hypoxia, Thermodesulfobacteriota, did not show a strong Mantel correlation with any single measured factor, though it displayed a weak negative trend with nitrate. This pattern indicates that the ascendancy of Thermodesulfobacteriota under hypoxia is not driven by a straightforward, strong correlation with low DO at the phylum level, but likely emerges from a complex interplay of factors.

**Figure 4:**
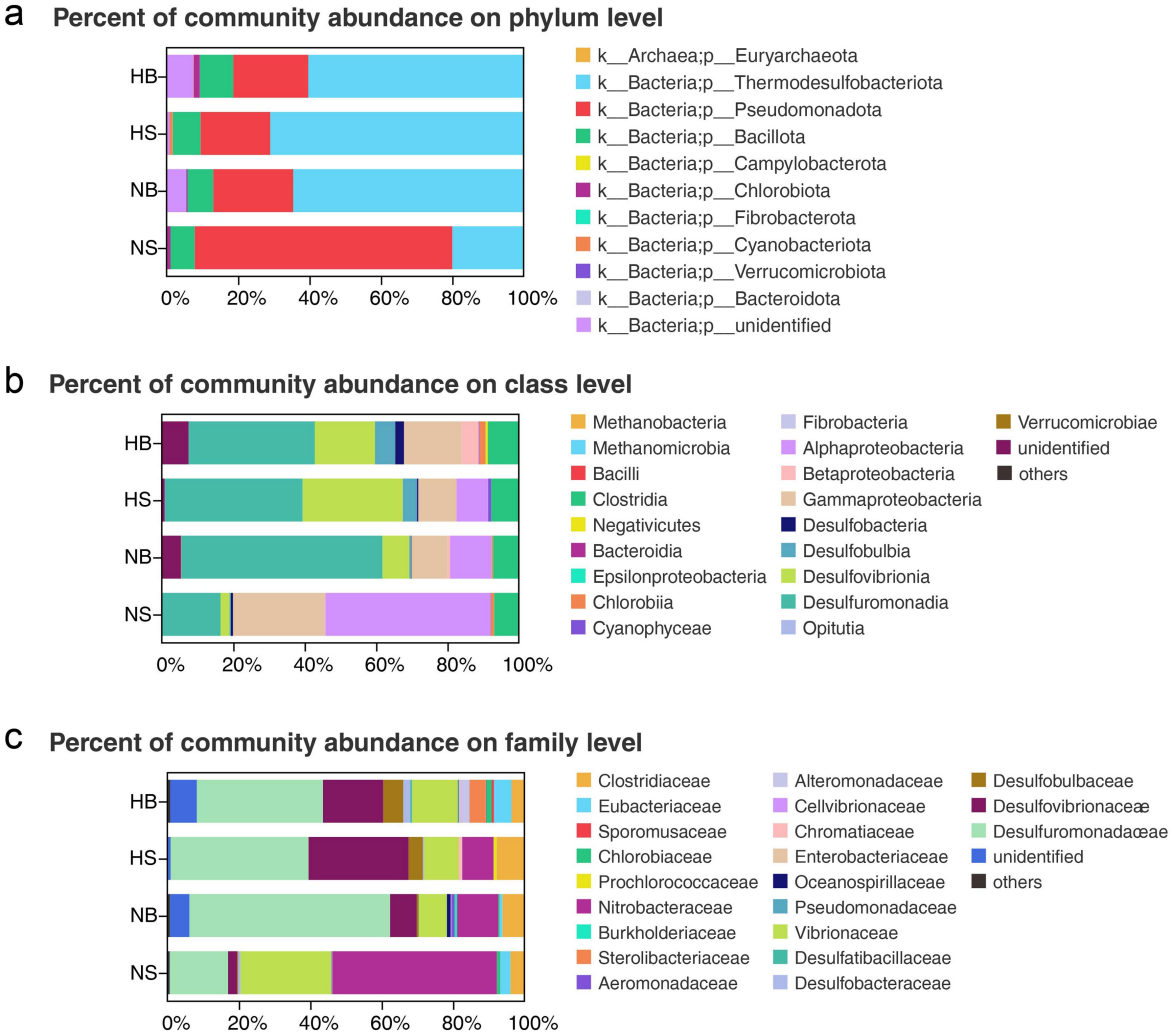
Diazotrophic community composition across hypoxic and non-hypoxic regions of the Changjiang Estuary at the **(a)** phylum, **(b)** class, and **(c)** family levels. Communities are shown for the hypoxic zone (all in the bottom, HB), surface layers above the hypoxic zone (HS), bottom layers in the non-hypoxic zone (NB), and surface layers in the non-hypoxic zone (NS).

A marked phylogenetic shift was observed between hypoxic and non-hypoxic waters. Thermodesulfobacteriota dominated most regions, comprising 60.34% of sequences in HB and maintaining high relative abundance in HS (70.99%) and NB (64.43%). In contrast, NS communities were dominated by Pseudomonadota (72.15%; Figure 4a and Supplementary Figure S3). At the class level, a total of 13, 13, 16, and 15 classes were identified in HB, HS, NB, and NS communities, respectively (Supplementary Figure S5). Desulfuromonadia and Gammaproteobacteria were consistently abundant across all regions (Figure 4b and Supplementary Figure S5). However, the class Desulfovibrionia was selectively enriched in hypoxic zones (HB and HS), whereas Alphaproteobacteria were more abundant in non-hypoxic regions (NB and NS; Table 2; Figure 4B). At the family level, a total of 34, 22, 37, and 35 families were detected in the HB, HS, NB, and NS, respectively (Supplementary Figure S5). Vibrionaceae and Desulfuromonadaceae were ubiquitous core groups across all samples. Hypoxia-selective enrichment was observed for Desulfovibrionaceae in HB and HS, while Nitrobacteraceae was dominant in NB and NS (Table 2; Figure 4c). Overall, diazotrophic community composition was primarily structured by oxygen conditions: hypoxic regions (HB and HS) shared both dominant groups and their order of dominance, whereas non-hypoxic regions (NB and NS) had the same dominant families but in a different order of relative abundance.

**Table 2:** Dominant groups in four regions.

Taxa	Rank	HB (Name; RA)	HS (Name; RA)	NB (Name; RA)	NS (Name; RA)
Class	Top 1	Desulfuromonadia; 35.37%	Desulfuromonadia; 38.61%	Desulfuromonadia; 56.31%	Alphaproteobacteria; 46.31%
	Top 2	Desulfovibrionia; 16.88%	Desulfovibrionia; 28.06%	Alphaproteobacteria; 11.56%	Gammaproteobacteria; 25.79%
	Top 3	Gammaproteobacteria; 16.16%	Gammaproteobacteria; 10.65%	Gammaproteobacteria; 10.14%	Desulfuromonadia; 16.33%
Family	Top 1	Desulfuromonadaceae; 35.35%	Desulfuromonadaceae; 38.60%	Desulfuromonadaceae; 56.19%	Nitrobacteraceae; 46.06%
	Top 2	Desulfovibrionaceae; 16.86%	Desulfovibrionaceae; 28.06%	Nitrobacteraceae; 11.55%	Vibrionaceae; 25.40%
	Top 3	Vibrionaceae; 12.76%	Vibrionaceae; 9.69%	Vibrionaceae; 7.79%	Desulfuromonadaceae; 16.30%

RA = Relative abundance; HB = Hypoxic-Bottom, represents hypoxic zone (all in the bottom, DO < 62.5 μmol L^−1^); HS = Hypoxic-Surface, represents surface layers above the hypoxic zone without hypoxia; NB = Non-hypoxic-Bottom, represents bottom layers in the non-hypoxic zone; NS = Non-hypoxic-Surface, represents surface layers in the non-hypoxic zone.

Further analysis highlighted hypoxia-associated taxonomic patterns. Archaeal sequences were detected exclusively in non-hypoxic regions, while Campylobacterota were restricted to hypoxic zones (Figure 4a). Moreover, the relative abundances of phyla Campylobacterota and Chlorobiota, as well as classes Negativicutes, Epsilonproteobacteria, Chlorobiia, Betaproteobacteria, Desulfobacteria, and Desulfobulbia were significantly higher in HB (≥ 50%) than in the other three regions (Figures 4a,b). Families such as Sporomusaceae (belongs to class Negativicutes), Sterolibacteriaceae (Betaproteobacteria), Alteromonadaceae (Gammaproteobacteria), and Enterobacteriaceae (Gammaproteobacteria) were nearly exclusive to HB region, with relative abundance ≥ 98% (Figure 4c). These findings underscore niche partitioning and suggest that metabolic specializations, particularly within Thermodesulfobacteriota underpin success under low-oxygen conditions. Conversely, the dominance of Pseudomonadota in oxic regions likely reflects competitive advantages under higher redox potentials.

### 3.4 Microbial biomarkers across oxygen gradients

**Figure 5:**
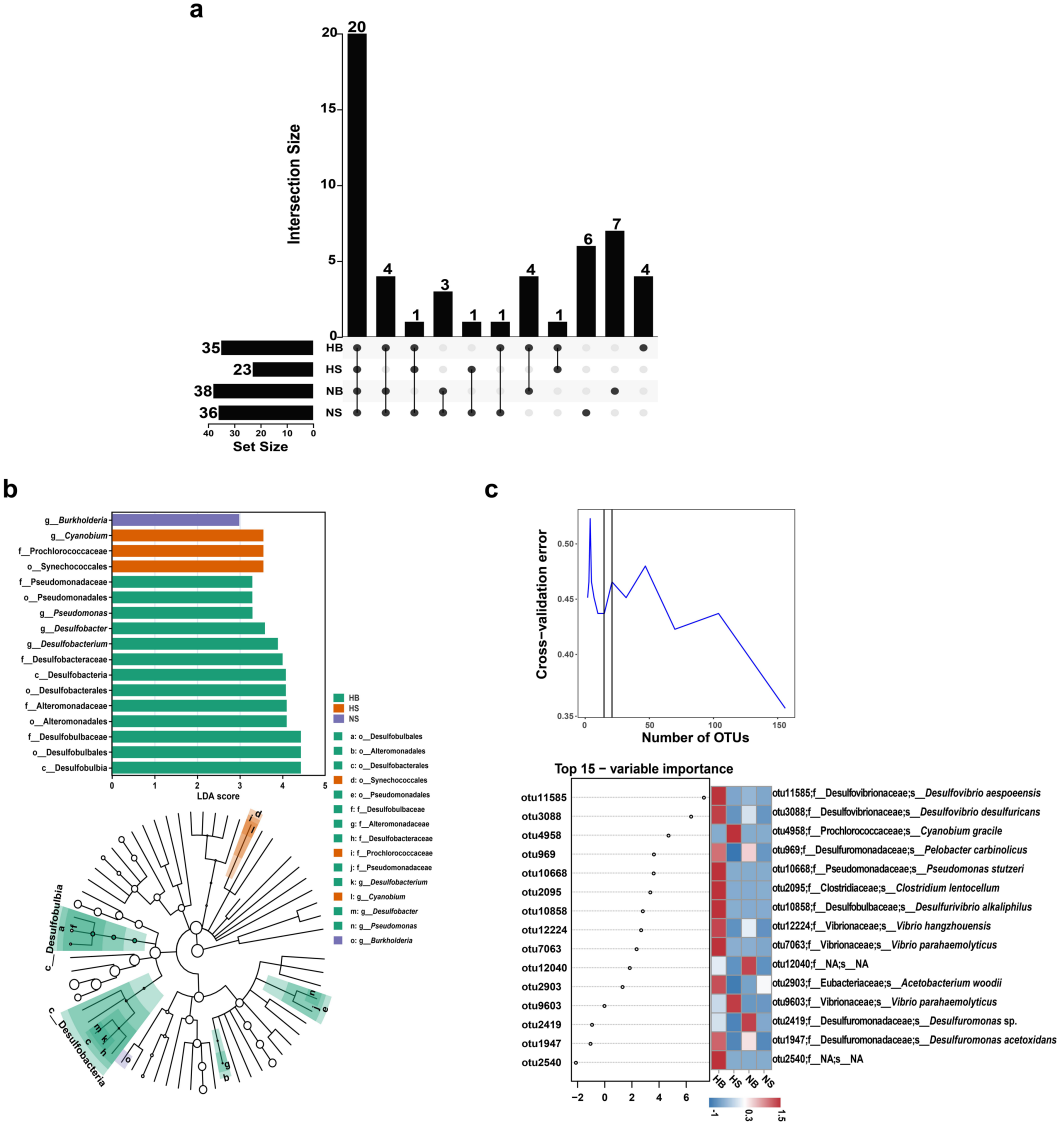
Oxygen-responsive biomarker identification in diazotrophic communities. **(a)** Family-level Upset diagram delineating shared and region-specific groups (Set Size: the total number of unique OTUs found exclusively within each individual group). **(b)** LEfSe-derived cladogram (linear discriminant analysis score ≥ 3, initiated at the OTU level) highlighting phylogenetically conserved hypoxia indicators. Colors of bars represent the groups with higher relative abundance (green: HB; orange: HS; purple: NS); Rings of taxonomic hierarchy tree, from inner to outer, represent phylum to genus, and the node sizes correspond to the average relative abundance of the groups; Colored nodes indicate groups showing significant differences (*p* < 0.05), with colors representing groups with highest relative abundance; The letters identify the names of groups. **(c)** Random Forest feature importance ranking of hypoxia-discriminatory OTUs, with mean decrease accuracy (MDA) quantifying predictive power. The heatmap shows the relative abundance in different regions.

To identify taxa indicative of hypoxic conditions, we applied multiple biomarker discovery methods. An Upset plot revealed 20 core families shared across all oxygen regions, along with 4, 7, and 6 families unique to HB, NB, and NS, respectively (Figure 5a). Notably, the hypoxia-exclusive families included the Thermodesulfobacteriota-associated Desulfonatronovibrionaceae and Desulfococcaceae, while non-hypoxic zones contained more unique families overall, but none belonging to Thermodesulfobacteriota (Figure 5a). These findings suggest that although Thermodesulfobacteriota were not uniformly dominant in HB, this region harbored unique lineages within the phylum that may serve as indicators of hypoxic conditions in the CE.

To further resolve key members across regions, we performed LEfSe analysis using LDA in combination with the K-W and Wilcoxon rank-sum tests to assess taxonomic differences at multiple levels. A total of 17 significantly taxonomic groups were identified across the oxygen gradient (LDA score ≥ 3, *p* < 0.05), of which 13 reached maximal relative abundance (green) in the HB (Figure 5b). Among these hypoxia-enriched taxa, eight belonged to Thermodesulfobacteriota, including classes Desulfobulbia and Desulfobacteria, orders Desulfobulbales and Desulfobacterales, families Desulfobulbaceae and Desulfobacteraceae, genera *Desulfobacterium* and *Desulfobacter*; Six (b, e, g, j, n and o in the tree) belonged to Pseudomonadota, including orders Alteromonadales and Pseudomonadales, families Alteromonadaceae and Pseudomonadaceae, genera *Pseudomonas* and *Burkholderia*; Three (d, i and l in the tree) belonged to Cyanobacteriota, namely order Synechococcales, family Prochlorococcaceae and genus *Cyanobium*. Notably, the two Thermodesulfobacteriota classes (Desulfobulbia and Desulfobacteria) also showed specific enrichment in the HB (relative abundance ≥ 50%) mentioned above. The family Alteromonadaceae (belongs to class Gammaproteobacteria), which showed near-exclusive localization in the HB (relative abundance ≥ 98%) was also emerged as a key differentiator in the LEfSe analysis.

Random forest modeling to identify OTUs with strong discriminatory power among HB, HS, NB, and NS communities. Based on 1,000 decision trees and 10-fold cross-validation, 12 biomarker OTUs with intergroup discriminatory ability were finally selected. Among them, seven belonged to Thermodesulfobacteriota and were dominated by sulfate-reducing specialists. *Desulfovibrio aespoeensis* (otu11585, Mean Decrease Accuracy = 7.6) was speculated to be the most important indicator, followed by *D. desulfuricans* (otu3088; MDA = 6.2), and the top four ranked OTUs all had peak abundance in the HB region (Figure 5c). Additional notable indicators from other phyla included *Vibro parahaemolyticus* (belongs to class Gammaproteobacteria) and *Clostridium lentocellum* (Clostridia). Overall, while some Thermodesulfobacteriota taxa occurred across all regions, their strong abundance gradients (e.g., HB: 60.34% vs. NS: 19.84%) and lineage-specific expansions (e.g., Desulfococcaceae) expansions highlight their value as robust bioindicators of redox conditions in the estuarine system.

### 3.5 Metagenomic validation of *nifH* abundance and metabolic potential

**Figure 6:**
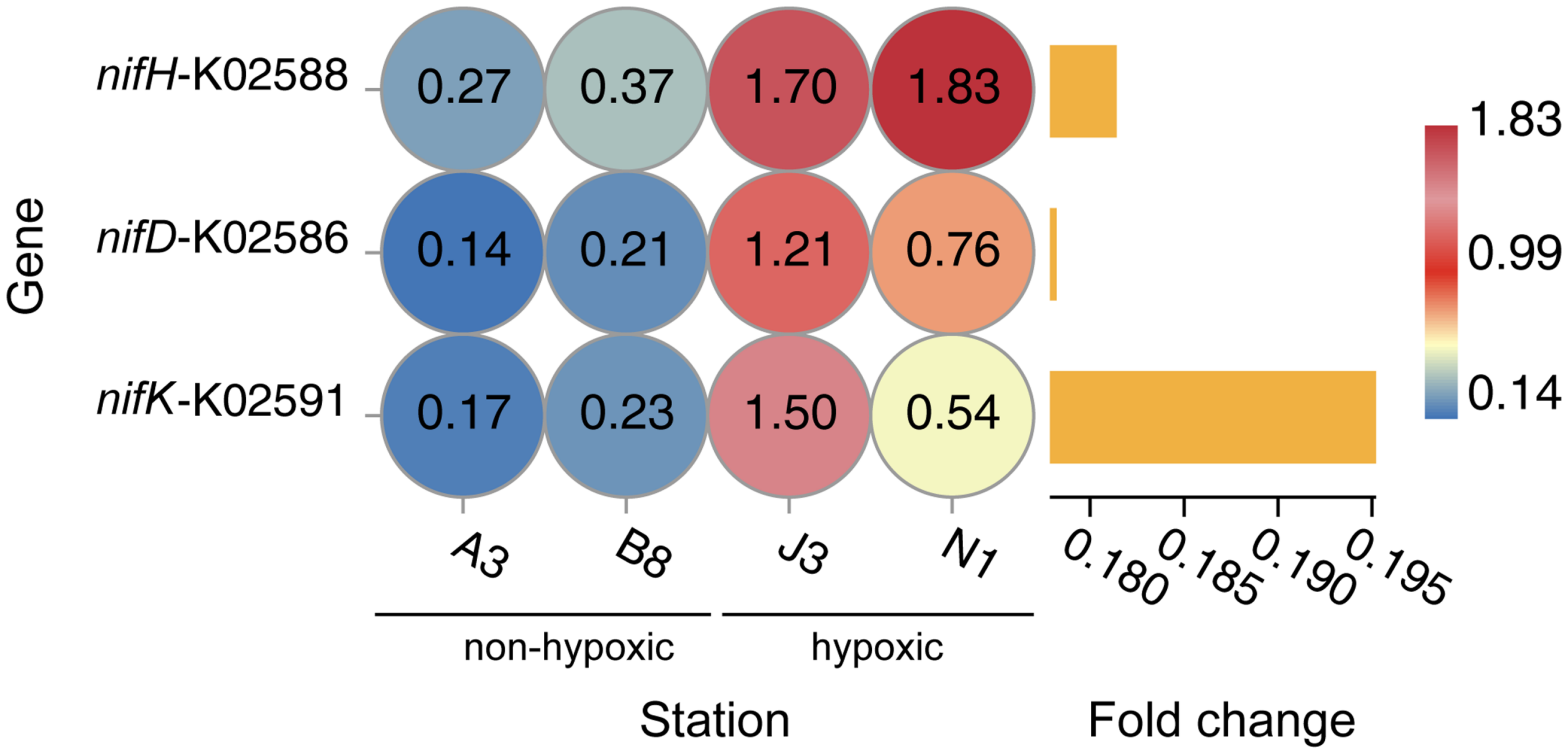
Relative abundance of the gene *nifKDH* (from metagenomic data) in pathway nitrogen fixation. The bar chart shows the fold change in mean relative abundance (non-hypoxic/hypoxic).

Metagenomic analysis independently validated the amplicon-based findings and further revealed the community’s metabolic potential of the community. The relative abundance of the nitrogenase gene *nifH* was significantly higher (5.5-fold) in hypoxic waters (3.53) compared to non-hypoxic waters (0.64; Figure 6). This result corroborated the amplicon-based community analysis, and confirmed the diverse of nitrogen-fixing microbes under low-oxygen conditions. Among the 11 high-quality Thermodesulfobacteriota MAGs reconstructed, the *nifH* gene was detected exclusively in MAG CRE_J3B_bin_5, which is affiliated with the family Desulfocapsaceae (Supplementary Table S2). This MAG exhibited 18.25-fold higher relative abundance in hypoxic zones compared to non-hypoxic environments (Supplementary Figure S6), indicating strong niche-specific adaptation.

Family-level functional profiling using METABOLIC-derived metabolic weight scores (MW-scores) identified Desulfocapsaceae (belongs to Thermodesulfobacteriota) as a key contributor to nitrogen fixation (Table 3). In addition to its primary nitrogen fixation capacity (MW-score = 34.7), this lineage demonstrated substantial potential for carbon fixation via the reverse TCA cycle (MW-score = 57.6) and sulfite reduction processes (MW-score = 25.9; Supplementary Table S3). These results suggest tight metabolic coupling between nitrogen, carbon, and sulfur cycles in response to hypoxia.

**Table 3:** Contributions of different families to N_2_ fixation genes.

Function	N-S-01: N_2_ fixation - *nifDK*||*vnfDKG*||*nifH*
Pirellulaceae	51.2
Desulfocapsaceae	34.7
Acidaminobacteraceae	12.7
UBA12075	1.5

### 3.6 Diazotroph community co-occurrence patterns

**Figure 7:**
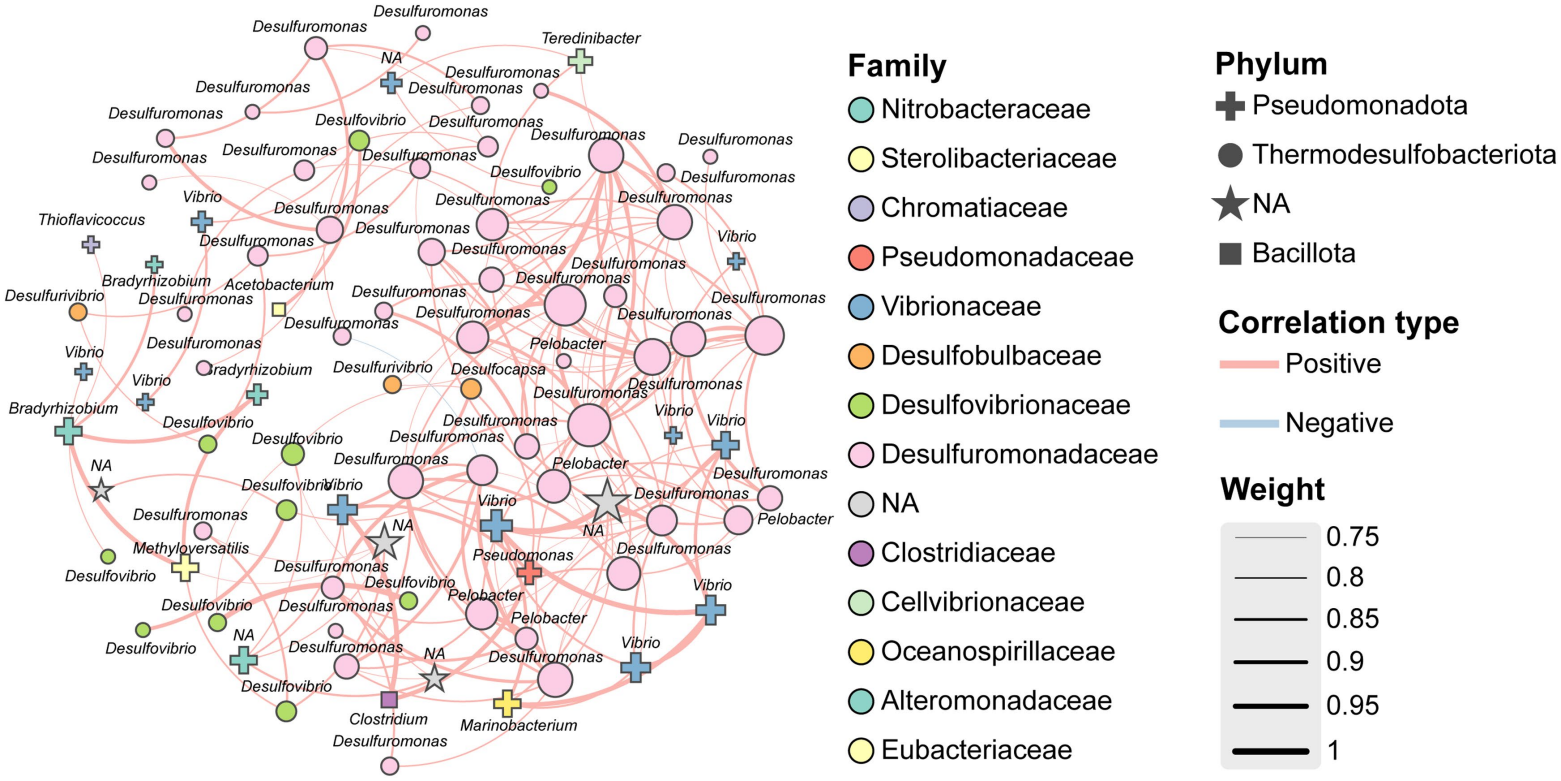
Co-occurrence patterns of diazotroph populations based on Spearman’s correlation analysis. Network nodes represent *nifH* OTUs, and the size of each node (degree) is proportional to the number of its associations; Different colors and shapes of nodes represent different families and phyla, respectively; Edge width reflects Spearman’s correlation strength (|r| > 0.7, *p* < 0.01).

A co-occurrence network was constructed to investigate interactions among key diazotrophic taxa. The resulting network consisted of 86 nodes (OTUs) connected by 186 edges, of which 185 (99.5%) represented positive correlations, and only one edge represented a negative correlation (Figure 7). This pattern indicated a strong overall tendency toward cooperative interactions within the community. Keystone OTUs were dominated by Thermodesulfobacteriota, which accounted for 68.6% of high-connectivity nodes (average degree = 4.33; Figure 7). Their topological dominance suggests that chemoautotrophic Thermodesulfobacteriota not only act as indicator groups for hypoxic zones in the CE, but may also play a central ecological role in linking diverse microbial community members. Their prevalence in key network positions highlights their potential importance in maintaining community stability and functional integrity, especially under low-oxygen conditions where cooperative metabolic interactions likely support diazotrophic resilience and biogeochemical cycling.

### 3.7 Relationships between environmental factors and key diazotrophic groups

**Figure 8:**
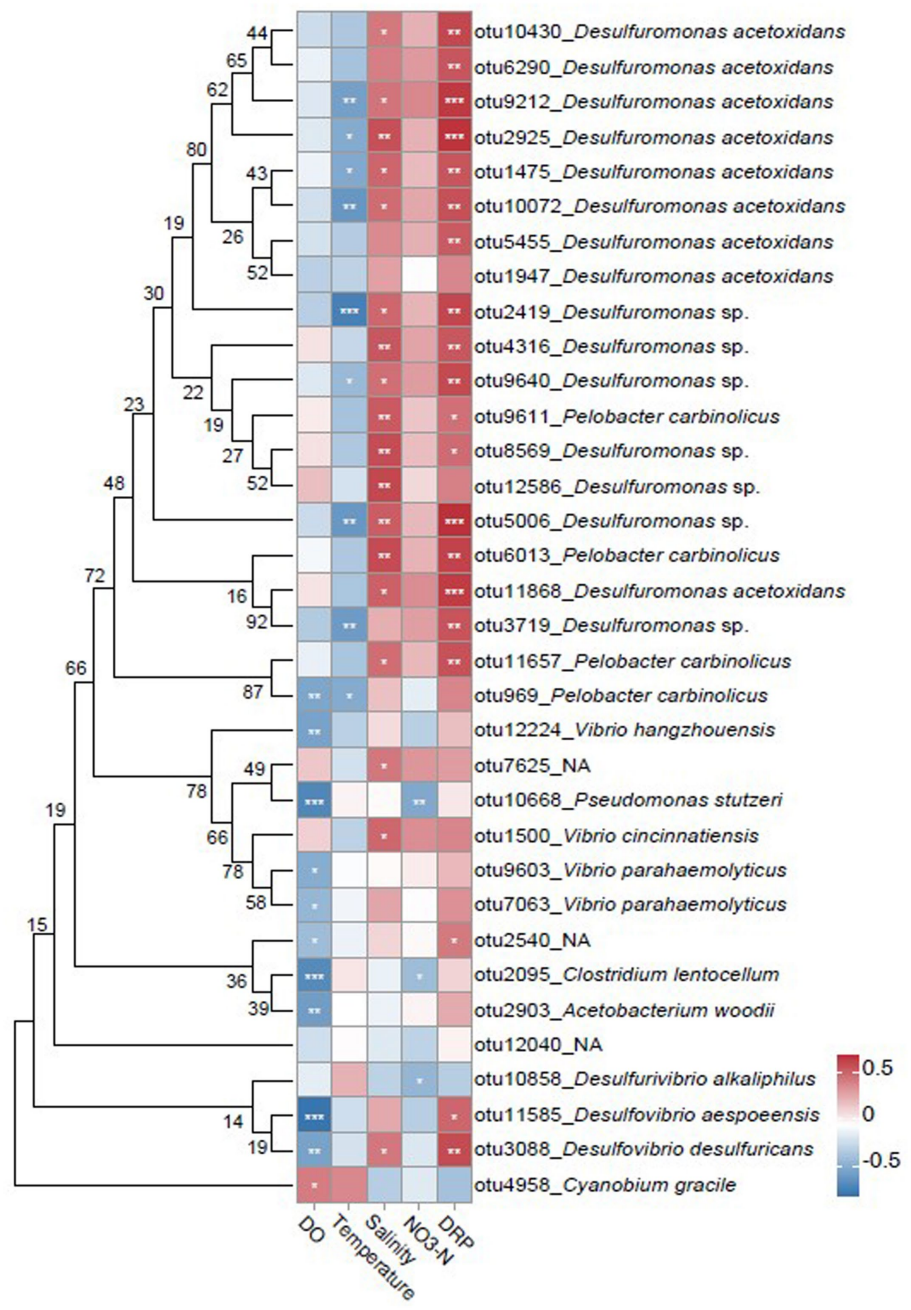
Spearman correlation heatmap of biomarker and keystone groups with environmental relationships. Color intensity reflects the correlation coefficient value (blue: negative; red: positive); Asterisks denote significance levels (*p < 0.05, **p < 0.01, ***p < 0.001); Phylogenetic tree was based on maximum likelihood (ML) analysis.

Spearman correlation analysis revealed significant associations between key diazotrophic groups and environmental variables (Figure 8). The relative abundance of most species, particularly within Thermodesulfobacteriota, was negatively correlated with DO concentration and temperature, but positively correlated with salinity, NO_3_^−^ and DRP concentrations (Figure 8). Specifically, Thermodesulfobacteriota showed significant positive correlations with both salinity and DRP (*p* < 0.05; Figure 8). Additionally, key species such as *Desulfovibrio aespoeensis* (otu11585), *Desulfovibrio desulfuricans* (otu3088) and *Pelobacter carbinolicus* (otu969) exhibited significant negative correlations with DO concentration (*p* < 0.05; Figure 8). In general, different taxa displayed distinct response patterns to environmental gradients, highlighting species-specific ecological adaptations within the estuarine system.

## 4 Discussion

### 4.1 Highly diverse diazotrophic communities in the hypoxic waters of the Changjiang estuary

Early studies suggested that diazotrophs preferentially thrive in warm, saline, oligotrophic surface waters of low-latitude regions (Dugdale et al., 1961; Capone et al., 1997). More recent research, however, has documented active nitrogen fixation in nutrient-rich systems, including upwelling zones, bays, estuaries, and river plumes (Voss et al., 2006; Ferrando and Scavino, 2015; Li et al., 2019), such as the Amazon Estuary (Subramaniam et al., 2008) and the Mekong Delta (Grosse et al., 2010). Additionally, diazotrophy is also no longer considered restricted to the euphotic zone, it has been observed in hypoxic waters, deep-sea environments, and surface sediments (Wu et al., 2019; Dong et al., 2022; Jiang et al., 2025). This study builds on these findings by characterizing diazotrophic diversity in the eutrophic, hypoxic zones of the CE and comparing it with adjacent oxic waters. Within the study area, DO concentrations in hypoxic zones fell to as low as 2.70 μmol L^−1^ (mean: 32.77 μmol L^−1^), significantly lower than in adjacent oxic waters (mean: 106.07 μmol L^−1^; Table 1). Metabarcoding analysis revealed markedly higher diazotrophic community diversity in hypoxic waters, with Shannon index values 1.2–1.7 times greater than those in oxic zones [*P* (HB-HS) = 0.017, *P* (HB-NB) = 0.031, and *P* (HB-NS) = 0.042; Figures 3b]. Metagenomic profiling further supported that *nifH* gene abundance was approximately 5.5-fold higher in hypoxic waters than in oxic regions (Figure 6), highlighting the increased diazotrophic biodiversity under hypoxic conditions.

A synthesis of previous studies indicates clear niche differentiation among diazotrophic guilds: cyanobacteria dominate warm tropical and subtropical oceans; heterotrophic Pseudomonadota prevail in nutrient-rich coastal waters and sediments; Archaea and Pseudomonadota dominate extreme environments such as deep-sea hydrothermal vents; Oligotrophic systems tend to host mixed cyanobacterial–Pseudomonadota consortia (Staal et al., 2003; Jayakumar et al., 2017; Martínez-Pérez et al., 2018). In this study, chemoautotrophic Thermodesulfobacteriota, which was previous misclassified within Pseudomonadota, dominated the hypoxic waters (with relative abundance of 58.93%), followed by heterotrophic Pseudomonadota (31.67%; Figure 2). These results are consistent with global patterns while refining taxonomic resolution and emphasizing the prominence of chemolithotrophic lineages in hypoxic, eutrophic estuaries.

The observed community structure appears to be shaped by multiple environmental and hydrological factors. Firstly, low oxygen tensions directly inhibit the electron transport chain activity in aerobic diazotrophs (Postgate, 1998), leading to drastic declines in phototrophic groups such as Cyanobacteriota (with relative abundance of 0.17%; Figure 4), consistent with global patterns in estuarine hypoxia (Rabalais et al., 2010). In contrast, anaerobic diazotrophs like sulfate-reducing bacteria (SRB-diazotrophs) use alternative electron acceptors (e.g., SO_4_^2−^, Fe^3+^) for energy (Slobodkin et al., 2013), supporting the dominance of Thermodesulfobacteriota and Pseudomonadota (Figure 4; Table 2). Secondly, N/P stoichiometric models suggest spatial coupling between nitrogen fixation and nitrogen loss (via denitrification and anammox; Deutsch et al., 2007), and hypoxia-enhanced denitrification depletes NO_3_^−^, which may intensify competition for nitrogen and promote diazotrophic diversity (Sarma et al., 2016). Our field measurements in this study found that NO_3_^−^ concentrations in hypoxic zones ranged from 0.30–22.50 μmol L^−1^ (mean: 10.07 μmol L^−1^), compared to 16.22 μmol L^−1^ in average in oxic zones (Table 1), the depletion of NO_3_^−^ may be one of the explanations for the highly diverse diazotrophic communities. Furthermore, the lower nitrate concentrations in bottom waters, attributed to high-salinity offshore water, suggest that water mass dynamics, rather than hypoxia alone, may influence nutrient distributions (Wu et al., 2022). This is supported by the high salinity reported in the study (Table 1). Thirdly, salinity-sulfur coupling may also contribute. Elevated salinity in hypoxic waters (mean: 32.41) and sediment-derived sulfate inputs supported SRB-diazotroph activity (Burgin et al., 2011; Severin et al., 2012; Figure 4). The strong positive correlations between dominant diazotrophs and both salinity and DRP (Figure 8) suggested adaptation to estuarine frontal zones and phosphorus mobilization processes, which also observed in the Amazon Estuary (Subramaniam et al., 2008) and Gulf of Mexico hypoxic zones (Sarma et al., 2016). Unlike the Amazon system, where low nutrient fluxes favor phototrophic dominance, the Changjiang Estuary’s steep redox gradients and high nutrient loading may favor chemoautotrophic guilds.

### 4.2 Origins of the hypoxic diazotrophic community: water mass intrusion versus *in situ* selection

Although coastal hypoxia often results from strong stratification and rapid microbial oxygen consumption, which may sustain microbial proliferation in hypoxic waters, the origin and proliferation of these diazotrophic communities remain unclear. The hypoxic zone in the CE is characterized by seasonal development (July–September), influenced by freshwater discharge, sediment resuspension, and intrusion of offshore saline water (Li et al., 2002; Dai et al., 2011). The high salinity and specific nutrient signatures in hypoxic bottom waters suggest that water mass dynamics, such as the intrusion of Kuroshio subsurface water, may deliver allochthonous microorganisms, including diazotrophs, into the estuary. Alternatively, these communities may originate from local sediments and proliferate in response to hypoxic conditions (Li et al., 2019; Lin et al., 2025; Sun et al., 2025). In this study, the significant co-variation of low DO and high salinity presents a challenge in attributing the observed community shift solely to hypoxia. The intrusion of a high-salinity water mass could introduce an allochthonous microbial community, offering an alternative explanation for our findings.

The multivariate statistical results reinforce the concept of multifactorial control in this dynamic ecosystem. The statistically significant yet weak separation (ANOSIM R = 0.169) between our pre-defined DO groups, along with the PERMANOVA result that DO uniquely explained only 9.69% of the community variance, underscores that hypoxia alone is an incomplete predictor. Meanwhile, it should also not be interpreted as a minor role for hypoxia, it underscores that the low-DO habitat is intrinsically linked to other physicochemical changes, affecting the observed diazotrophic community structure. Our DO-based grouping effectively captures a syndromic environmental state characteristic of the CE summer, which is defined not only by low oxygen but also by high salinity, specific nutrient regimes, and distinct water mass origins (e.g., the intrusion of Kuroshio subsurface water; Wu et al., 2022). The significant independent contribution of salinity (6.42%) highlights that the observed community shift could be attributed to both the direct physiological effects of low oxygen and the introduction of a microbial community adapted to the saline, nutrient-replete conditions of the intruding water mass (Song et al., 2022). This is a typical characteristic of stratified estuaries, where physical water mass dynamics and chemical gradients are inextricably linked, collectively shaping the microbial assemblage (Song et al., 2022). Therefore, the hypoxic zone in estuaries supports a unique diazotrophic assemblage that is a product of the synergistic pressures of low DO and its associated environmental covariates.

While our data cannot fully rule out this contribution, several lines of evidence converge to suggest that *in situ* environmental selection, particularly by low DO, is a primary driver of the observed patterns, and that water mass intrusion likely delivers pre-adapted taxa which then proliferate. First, although PERMANOVA indicates that both DO and salinity are significant independent factors (uniquely explaining 9.69 and 6.42% of the variance, respectively; Supplementary Table S1), the specific, hypoxia-driven enrichment of Thermodesulfobacteriota is striking. Its relative abundance increases approximately threefold from non-hypoxic surface waters (19.84%) to the hypoxic bottom (60.34%; Figure 4). A mere physical introduction via water mass intrusion would not necessarily cause such a dramatic relative dominance within the community unless the introduced taxa were also simultaneously experiencing strong *in situ* selective pressure that favored them over resident and other introduced species (Thompson et al., 2020). Second, the reconstructed metabolic pathways for the dominant Thermodesulfobacteriota (e.g., within Desulfocapsaceae) reveal a genetic capacity not just for nitrogen fixation, but for a coherent physiological strategy tailored to hypoxic, sulfidic environments (Supplementary Table S3). This genetic toolkit is a clear signature of adaptation to the very conditions that define the hypoxic zone. It is highly improbable that the possession of these specific adaptive traits and their consequent manifestation in population dominance is a mere coincidence following passive transport. The congruence between their genomic capacity and the environmental conditions strongly indicates that low DO acts as a stringent environmental filter, selectively favoring taxa with these pre-existing metabolic capabilities, allowing them to outcompete others and proliferate. Finally, the prominence of SRB like Thermodesulfobacteriota as diazotrophs is a recurring theme in globally distributed oxygen-deficient zones and sediments, underscoring a conserved ecological function that is intrinsically linked to low-oxygen habitats rather than to a specific water mass (Zhao et al., 2024). Therefore, we propose a synthesized model: the intrusion of high-salinity water may serve as a vector, delivering a seed population of microbes pre-adapted to marine and potentially suboxic conditions. However, the ultimate dominance of Thermodesulfobacteriota is a product of *in situ* selection, where the pervasive pressure of hypoxia selectively amplifies those taxa whose metabolic architecture is best suited to exploit this challenging environment.

This study is design to use DO concentration as a primary medium to group and discern the dynamics of diazotrophic communities. Our findings underscore that the resultant community structure is a consequence of the synergistic pressures within the hypoxic syndromic environment. Future work that simultaneously tracks the trajectories of distinct water masses and measures a broader suite of *in situ* biogeochemical rates will be crucial to quantitatively partition the influence of physical transport versus local conditions, and to fully resolve the interactive effects of the multiple environmental drivers.

### 4.3 Ecological and biogeochemical implications of Thermodesulfobacteriota

In our study site, environmental selection pressures reduced the prevalence of traditional aerobic diazotrophs, creating conditions favorable to anaerobic lineages. Notably, chemoautotrophic sulfate-reducing Thermodesulfobacteriota demonstrated a clear ecological advantage in the hypoxic zones of the CE (Figure 4). Phylogenomic analysis has revealed distinct genomic features in Thermodesulfobacteriota, such as sulfur metabolism gene clusters (*dsrAB-nifH*) and CRISPR-Cas antiviral systems, which support their recognition as an independent phylum now (Waite et al., 2020). Previous studies have confirmed active nitrogen fixation by Thermodesulfobacteria in estuarine rhizosphere sediments (Bertics and Ziebis, 2010; Wang et al., 2024). While their diazotrophic role in aquatic systems remains poorly defined, our metagenomic analyses revealed nitrogen fixation potential within the family Desulfocapsaceae (MW-score = 34.7; Table 3; Supplementary Table S3).

Beyond chemolithoautotrophy, heterotrophic metabolism may also contribute to diazotrophy in this organic-rich system. The CE receives substantial inputs of particulate and dissolved organic matter (Gao et al., 2012; Bao et al., 2015), which could fuel heterotrophic nitrogen fixation. The detection of heterotrophic Pseudomonadota (31.67%) and facultative anaerobes like Vibrio and Clostridium (Figure 5c) supports this view. Thus, both autotrophic and heterotrophic pathways likely coexist, together sustaining the high diversity observed in hypoxic waters.

Environmental selection appears to drive Thermodesulfobacteriota toward a hierarchical metabolic strategy, encompassing energy acquisition, carbon–nitrogen co-assimilation, and oxidative stress resistance, establishing their dominance in hypoxic environments as biogeochemical engines. For energy metabolism, they utilize diverse electron transport chains (e.g., sulfate and iron reduction, hydrogen oxidation), facilitating adaptation to dynamic redox conditions (Slobodkin et al., 2013). Under hypoxia, nitrogenase activity increases by approximately 2.3-fold compared to aerobic conditions (Krukenberg et al., 2016). For carbon-nitrogen co-assimilation, the Wood–Ljungdahl pathway (MW-score = 57.6) provided approximately 1.5 times greater carbon fixation efficiency than the Calvin cycle (Supplementary Table S3). Concurrent nitrogen fixation supplemented bioavailable nitrogen (Figure 6), forming an autotrophic loop critical in dissolved organic carbon (DOC)-depleted bottom waters (Krukenberg et al., 2016). This mechanism may help explain the high rates of sedimentary organic carbon burial in the CE hypoxic zone: sulfur-oxidizing nitrogen fixation by Thermodesulfobacteriota could enhance organic matter preservation by mitigating H_2_S toxicity (Jørgensen and Parkes, 2010). Antioxidant defenses, such as carotenoid synthesis and alkyl hydroperoxide reductase production, further mitigate oxidative stress during sulfidogenesis (Slobodkin et al., 2013; Waite et al., 2020; Wang et al., 2024). These adaptations were evident in our metabarcoding data, which showed hypoxia-specific enrichment of families such as Desulfonatronovibrionaceae and Desulfococcaceae (Figure 5A), alongside redox-driven shifts in more generalist groups (Figure 5).

Co-occurrence network analysis revealed Thermodesulfobacteriota as keystone taxa, occupying 68.60% of highly connected nodes (Figure 7). Their ecological centrality was evident: removal of Desulfuromonadaceae reduced network modularity by 75.3% (edges dropped from 186 to 46), suggesting potential for cascading collapse. Their prominence likely reflects syntrophic interactions and metabolic exchange. For instance, Thermodesulfobacteriota may form hydrogenotrophic partnerships with methanogens (e.g., *Methanosaeta*), releasing H_2_ to fuel methanogenesis while reducing H_2_ partial pressure to sustain thermodynamic feasibility (Krukenberg et al., 2016). However, our *nifH*-targeted sequencing detected minimal methanogenic presence (belonging to Euryarchaeota; Figure 2), with negligible network associations (Figure 7). Additionally, electron shuttle sharing (e.g., riboflavin) may facilitate Fe^3+^ reduction and nitrogenase activation (Waite et al., 2020). Sulfur disproportionation could also generate elemental sulfur (S^0^), fueling sulfur oxidizers (e.g., *Thiobacillus*) and strengthening S–N coupling (Bertics et al., 2013; Fulweiler et al., 2013; Figure 7). Meanwhile, H_2_S byproducts may stimulate phosphorus release, generating a positive S–P–N feedback loop (supported by observed correlations between DRP and Thermodesulfobacteriota abundance; Figure 8). The overwhelming dominance of positive correlations (185 vs. 1 negative) underscores the importance of cooperative interactions for hypoxia adaptation.

Importantly, Thermodesulfobacteriota may translate their metabolic capabilities into ecosystem services. For example, sulfate reduction consumes protons, alleviating sediment acidification, while nitrogen fixation partially offsets nitrogen losses in hypoxic systems (Rabalais et al., 2010; Sarma et al., 2016). However, H_2_S production (3.2 mmol m^−2^ d^−1^) approaches benthic LC_50_ toxicity thresholds (approximately 2.1 μM), potentially inhibiting the recruitment of sensitive fauna such as polychaete larvae (Reis et al., 1992; Joye and Hollibaugh, 1995; Yang et al., 2019). Thus, Thermodesulfobacteriota function as both key biogeochemical drivers and potential ecological risk agents, highlighting the need for monitoring sulfate concentrations and balancing nitrogen retention against sulfur toxicity.

While SRB are recognized diazotrophs (Nielsen et al., 2001; Fulweiler et al., 2013; Gier et al., 2016; Shiau et al., 2017) capable of coupling sulfate reduction with nitrogen fixation (Hou et al., 2018), and our multi-omics approach provides strong correlative evidence for nitrogen fixation within Thermodesulfobacteriota under hypoxia, it is crucial to note that these findings point to a dominant potential rather than a directly measured activity. The presence and abundance of the *nifH* gene are robust proxies, but they do not unequivocally demonstrate *in situ* nitrogen fixation rates. To conclusively confirm the inferred ecological role of Thermodesulfobacteriota as active diazotrophs in the CE hypoxic zone, future studies should integrate direct rate measurements, such as the ^15^N₂ stable isotope assimilation assay (Großkopf et al., 2012; Liu et al., 2020), with molecular activity assessments like reverse transcription quantitative PCR (RT-qPCR; Chen et al., 2019) targeting *nifH* mRNA. Such an integrated framework will be essential to bridge the gap between genetic potential and realized ecosystem function, providing a more definitive understanding of the drivers and magnitudes of nitrogen fixation in coastal hypoxic systems.

## 5 Conclusion

In the context of the hypoxia event in the Changjiang Estuary, this study characterized the structure of diazotrophic communities in hypoxic waters, compared their differences with non-hypoxic waters, and examined complex species-species interactions alongside environmental influences on key groups. Notably, diazotrophic biodiversity was found highly diverse under hypoxic conditions, with non-cyanobacterial diazotrophs emerging as the dominant groups in coastal waters. Among them, chemoautotrophic Thermodesulfobacteriota were particularly prominent, as revealed by *nifH* gene amplicon sequencing and metagenomic analysis. Furthermore, LEfSe analysis, random forest modeling, and network analysis identified significant bioindicator and keystone groups distinguishing hypoxic from non-hypoxic environments, with Thermodesulfobacteriota species serving as important indicators and network hubs. Environmental drivers including low DO concentrations, elevated salinity, increased DRP concentrations, and nitrate depletion appeared to promote potential nitrogen fixation under hypoxia. Findings from both amplicon and metagenomic analyses consistently suggested that coastal hypoxia supports diverse diazotrophic biodiversity and potential biogeochemical activity compared to oxygenated waters, with Thermodesulfobacteriota potentially playing a dual role in both nitrogen and carbon–sulfur cycling. The insights gained from this study advance our understanding of diazotrophs in the Changjiang Estuary. However, it is important to acknowledge that relative abundance data alone do not fully reflect the actual contribution of different groups to nitrogen fixation. To move beyond correlation and confirm the active ecological role of Thermodesulfobacteriota, future research must prioritize direct *in situ* measurements, such as ^15^N₂ assimilation assays coupled with quantification of *nifH* gene expression. This will be crucial to better assess the functional contributions of specific diazotrophic groups.

## Data Availability

The amplicon sequencing results (raw data) have been submitted to NCBI (https://www.ncbi.nlm.nih.gov), BioProject number is PRJNA1183438. The metagenomic sequencing data can be viewed in NODE (http://www.biosino.org/node), project ID OEP00000802. The physical and chemical data for this paper are freely available online through Figshare at: https://doi.org/10.6084/m9.figshare.28877102.Tools for data analysis were obtained from GitHub: Fastp v0.23.2 (https://github.com/OpenGene/fastp/); MEGAHIT v1.2.9 (https://github.com/voutcn/megahit); BBMap v38.87 (https://github.com/BioInfoTools/BBMap/blob/master/sh/rqcfilter.sh). Figures were made with Ocean Data View version 5.6.5 (http://odv.awi.de; Schlitzer, 2018) and R software version 3.6.1 (https://cran.r-project.org/bin/windows/base/; R Core Team, 2019).

## Footnotes

1
https://github.com/OpenGene/fastp/

2
https://github.com/voutcn/megahit

3
https://github.com/BioInfoTools/BBMap/blob/master/sh/rqcfilter.sh

4
http://huttenhower.sph.harvard.edu/galaxy/

## References

[ref1] Banerjee S. Schlaeppi K. van der Heijden M. G. A. (2018). Keystone taxa as drivers of microbiome structure and functioning. Nat. Rev. Microbiol. 16, 567–576. doi: 10.1038/s41579-018-0024-1, 29789680

[ref2] Bao H. Y. Wu Y. Zhang J. (2015). Spatial and temporal variation of dissolved organic matter in the Changjiang: fluvial transport and flux estimation. J. Geophys. Res. Biogeosci. 120, 1870–1886. doi: 10.1002/2015jg002948

[ref3] Bertics V. J. Löscher C. R. Salonen I. Dale A. W. Gier J. Schmitz R. A. . (2013). Occurrence of benthic microbial nitrogen fixation coupled to sulfate reduction in the seasonally hypoxic Eckernforde Bay, Baltic Sea. Biogeosciences 10, 1243–1258. doi: 10.5194/bg-10-1243-2013

[ref4] Bertics V. J. Ziebis W. (2010). Bioturbation and the role of microniches for sulfate reduction in coastal marine sediments. Environ. Microbiol. 12, 3022–3034. doi: 10.1111/j.1462-2920.2010.02279.x, 20561019

[ref5] Blaxter M. Mann J. Chapman T. Thomas F. Whitton C. Floyd R. . (2005). Defining operational taxonomic units using DNA barcode data. Philos. Trans. Royal Soc. B Biol. Sci. 360, 1935–1943. doi: 10.1098/rstb.2005.1725, 16214751 PMC1609233

[ref6] Bokulich N. A. Subramanian S. Faith J. J. Gevers D. Gordon J. I. Knight R. . (2013). Quality-filtering vastly improves diversity estimates from Illumina amplicon sequencing. Nat. Methods 10, 57–U11. doi: 10.1038/Nmeth.2276 23202435 PMC3531572

[ref7] Bowers R. M. Kyrpides N. C. Stepanauskas R. Harmon-Smith M. Doud D. Reddy T. B. K. . (2017). Minimum information about a single amplified genome (MISAG) and a metagenome-assembled genome (MIMAG) of bacteria and archaea. Nat. Biotechnol. 35, 725–731. doi: 10.1038/nbt.3893, 28787424 PMC6436528

[ref8] Burgin A. J. Yang W. H. Hamilton S. K. Silver W. L. (2011). Beyond carbon and nitrogen: how the microbial energy economy couples elemental cycles in diverse ecosystems. Front. Ecol. Environ. 9, 44–52. doi: 10.1890/090227

[ref9] Capone D. G. Burns J. A. Montoya J. P. Subramaniam A. Mahaffey C. Gunderson T. . (2005). Nitrogen fixation by *Trichodesmium* spp.: an important source of new nitrogen to the tropical and subtropical North Atlantic Ocean. Glob. Biogeochem. Cycles 19:331. doi: 10.1029/2004gb002331

[ref10] Capone D. G. Zehr J. P. Paerl H. W. Bergman B. Carpenter E. J. (1997). *Trichodesmium*, a globally significant marine cyanobacterium. Science 276, 1221–1229. doi: 10.1126/science.276.5316.1221

[ref11] Caporaso J. G. Kuczynski J. Stombaugh J. Bittinger K. Bushman F. D. Costello E. K. . (2010). QIIME allows analysis of high-throughput community sequencing data. Nat. Methods 7, 335–336. doi: 10.1038/nmeth.f.303, 20383131 PMC3156573

[ref12] Chaumeil P. A. Mussig A. J. Hugenholtz P. Parks D. H. (2022). GTDB-Tk v2: memory friendly classification with the genome taxonomy database. Bioinformatics 38, 5315–5316. doi: 10.1093/bioinformatics/btac672, 36218463 PMC9710552

[ref13] Chen M. M. Lu Y. Y. Jiao N. Z. Tian J. W. Kao S. J. Zhang Y. (2019). Biogeographic drivers of diazotrophs in the western Pacific Ocean. Limnol. Oceanogr. 64, 1403–1421. doi: 10.1002/lno.11123

[ref14] Dai Z. J. Du J. Z. Zhang X. L. Su N. Li J. F. (2011). Variation of riverine material loads and environmental consequences on the Changjiang (Yangtze) estuary in recent decades (1955-2008). Environ. Sci. Technol. 45, 223–227. doi: 10.1021/es103026a, 21128630

[ref15] Deutsch C. Sarmiento J. L. Sigman D. M. Gruber N. Dunne J. P. (2007). Spatial coupling of nitrogen inputs and losses in the ocean. Nature 445, 163–167. doi: 10.1038/nature05392, 17215838

[ref16] Dixon P. (2003). VEGAN, a package of R functions for community ecology. J. Veg. Sci. 14, 927–930. doi: 10.1658/1100-9233(2003)014[0927:Vaporf]2.0.Co;2

[ref17] Dong X. Y. Zhang C. W. Peng Y. Y. Zhang H. X. Shi L. D. Wei G. S. . (2022). Phylogenetically and catabolically diverse diazotrophs reside in deep-sea cold seep sediments. Nat. Commun. 13:4885. doi: 10.1038/s41467-022-32503-w, 35985998 PMC9391474

[ref18] Dugdale R. C. Menzel D. W. Ryther J. H. (1961). Nitrogen fixation in the Sargasso Sea. Deep-Sea Res. 7, 297–300. doi: 10.1016/0146-6313(61)90051-X, 41203699

[ref19] Edgar R. C. (2010). Search and clustering orders of magnitude faster than BLAST. Bioinformatics 26, 2460–2461. doi: 10.1093/bioinformatics/btq461, 20709691

[ref20] Farnelid H. Andersson A. F. Bertilsson S. Abu Al-Soud W. Hansen L. H. Sorensen S. . (2011). Nitrogenase gene amplicons from global marine surface waters are dominated by genes of non-cyanobacteria. PLoS One 6:9223. doi: 10.1371/journal.pone.0019223 PMC3084785 21559425

[ref21] Ferrando L. Scavino A. F. (2015). Strong shift in the diazotrophic endophytic bacterial community inhabiting rice (*Oryza sativa*) plants after flooding. FEMS Microbiol. Ecol. 91:fiv104. doi: 10.1093/femsec/fiv104, 26324852

[ref22] Fulweiler R. W. Brown S. M. Nixon S. W. Jenkins B. D. (2013). Evidence and a conceptual model for the co-occurrence of nitrogen fixation and denitrification in heterotrophic marine sediments. Mar. Ecol. Prog. Ser. 482, 57–68. doi: 10.3354/meps10240

[ref23] Gallon J. R. (2001). N_2_ fixation in phototrophs: adaptation to a specialized way of life. Plant Soil 230, 39–48. doi: 10.1023/A:1004640219659

[ref24] Gao L. Li D. J. Zhang Y. W. (2012). Nutrients and particulate organic matter discharged by the Changjiang (Yangtze River): seasonal variations and temporal trends. J. Geophys. Res. Biogeosciences 117, 1–16. doi: 10.1029/2012jg001952

[ref25] Gier J. Sommer S. Löscher C. R. Dale A. W. Schmitz R. A. Treude T. (2016). Nitrogen fixation in sediments along a depth transect through the Peruvian oxygen minimum zone. Biogeosciences 13, 4065–4080. doi: 10.5194/bg-13-4065-2016

[ref26] Grasshoff K. Ehrhardt M. (1999). Methods of seawater analysis. 3rd Edn. Cham: Springer.

[ref27] Grosse J. Bombar D. Hai N. D. Lam N. N. Voss M. (2010). The Mekong River plume fuels nitrogen fixation and determines phytoplankton species distribution in the South China Sea during low- and high-discharge season. Limnol. Oceanogr. 55, 1668–1680. doi: 10.4319/lo.2010.55.4.1668

[ref28] Großkopf T. Mohr W. Baustian T. Schunck H. Gill D. Kuypers M. M. . (2012). Doubling of marine dinitrogen-fixation rates based on direct measurements. Nature 488, 361–364. doi: 10.1038/nature11338, 22878720

[ref29] Guimera R. Nunes Amaral L. A. (2005). Functional cartography of complex metabolic networks. Nature 433, 895–900. doi: 10.1038/nature03288, 15729348 PMC2175124

[ref30] Hou L. J. Wang R. Yin G. Y. Liu M. Zheng Y. L. (2018). Nitrogen fixation in the intertidal sediments of the Yangtze estuary: occurrence and environmental implications. J Geophys Res Biogeosci 123, 936–944. doi: 10.1002/2018jg004418

[ref31] Jana T. Lam-Tung N. A. von H. Quang M. B. (2016). W-IQ-tree: a fast online phylogenetic tool for maximum likelihood analysis. Nucleic Acids Res. 44, W232–W235. doi: 10.1093/nar/gkw256 27084950 PMC4987875

[ref32] Jayakumar A. Chang B. N. X. Widner B. Bernhardt P. Mulholland M. R. Ward B. B. (2017). Biological nitrogen fixation in the oxygen-minimum region of the eastern tropical North Pacific Ocean. ISME J. 11, 2356–2367. doi: 10.1038/ismej.2017.97 28742073 PMC5607377

[ref33] Jiang Q. Y. Cao L. Han Y. C. Li S. J. Zhao R. Zhang X. L. . (2025). Cold seeps are potential hotspots of deep-sea nitrogen loss driven by microorganisms across 21 phyla. Nat. Commun. 16:1646. doi: 10.1038/s41467-025-56774-1, 39952920 PMC11828985

[ref34] Jørgensen B. B. Parkes R. J. (2010). Role of sulfate reduction and methane production by organic carbon degradation in eutrophic fjord sediments (Limfjorden, Denmark). Limnol. Oceanogr. 55, 1338–1352. doi: 10.4319/lo.2010.55.3.1338

[ref35] Joye S. B. Hollibaugh J. T. (1995). Influence of sulfide inhibition of nitrification on nitrogen regeneration in sediments. Science 270, 623–625. doi: 10.1126/science.270.5236.623

[ref36] Karl D. M. Letelier R. M. (2008). Nitrogen fixation-enhanced carbon sequestration in low nitrate, low chlorophyll seascapes. Mar. Ecol. Prog. Ser. 364, 257–268. doi: 10.3354/meps07547

[ref37] Karl D. Michaels A. Bergman B. Capone D. Carpenter E. Letelier R. . (2002). Dinitrogen fixation in the world's oceans. Biogeochemistry 57, 47–98. doi: 10.1023/A:1015798105851

[ref9001] Krukenberg V. Harding K. Richter M. Glöckner F. K. Gruber-Vodicka H. R. Adam B. . (2016). Candidatus Desulfofervidus auxilii, a hydrogenotrophic sulfate-reducing bacterium involved in the thermophilic anaerobic oxidation of methane. Environmental Microbiology 18, 3073–3091. doi: 10.1111/1462-2920.13283 26971539

[ref39] Li D. W. Chen J. F. Wang B. Jin H. Y. Shou L. Lin H. . (2024). Hypoxia triggered by expanding river plume on the East China Sea inner shelf during flood years. J. Geophys. Res. Oceans 129:1299. doi: 10.1029/2024JC021299

[ref40] Li D. Liu J. Zhang R. Chen M. Yang W. Li J. . (2019). N_2_ fixation impacted by carbon fixation via dissolved organic carbon in the changing Daya bay, South China Sea. Sci. Total Environ. 674, 592–602. doi: 10.1016/j.scitotenv.2019.04.176, 31022548

[ref41] Li D. J. Zhang J. Wu Y. Liang J. Huang D. J. (2002). Oxygen deficiency in Changjiang estuary (in Chinese). Sci. China Ser. D Earth Sci. 32, 687–694. doi: 10.3321/j.issn:1006-9267.2002.08.009

[ref42] Liaw A. Wiener M. (2002). Classification and regression by randomforest. R News 2, 18–22.

[ref43] Lin Y. Wang Y. Zhu J. Qiu C. Wu H. (2025). Extreme heatwave affects the saltwater intrusion and river plume extension in the Changjiang River estuary. J. Geophys. Res. Oceans 130:287. doi: 10.1029/2024JC022287

[ref44] Liu J. Y. Cui Z. S. Luan X. Wang Z. L. Zhang X. L. (2024). Expanding oxygen minimum zones in the northern Indian Ocean predicted by hypoxia-related bacteria. Front. Mar. Sci. 11:6306. doi: 10.3389/fmars.2024.1396306

[ref45] Liu J. J. Du P. Zeng J. N. Chen Q. Z. Shou L. Liao Y. B. . (2013). The ecological distributions of N, P utilizing bacteria and heterotrophic bacteria in the moderate hypoxia zone of the Changjiang estuary. J. Ocean Univ. China 12, 589–598. doi: 10.1007/s11802-013-2210-0

[ref46] Liu M. Xiao T. Wu Y. Zhou F. Huang H. Q. Bao S. X. . (2012). Temporal distribution of bacterial community structure in the Changjiang estuary hypoxia area and the adjacent East China Sea. Environ. Res. Lett. 7, 1–11. doi: 10.1088/1748-9326/7/2/025001

[ref47] Liu J. X. Zhou L. B. Li J. J. Lin Y. Y. Ke Z. X. Zhao C. Y. . (2020). Effect of mesoscale eddies on diazotroph community structure and nitrogen fixation rates in the South China Sea. Reg. Stud. Mar. Sci. 35:101106. doi: 10.1016/j.rsma.2020.101106

[ref48] Löscher C. Mohr W. Bange H. W. Canfield D. E. (2020). No nitrogen fixation in the bay of Bengal? Biogeosciences 17, 851–864. doi: 10.5194/bg-17-851-2020

[ref49] Lu B. B. Sun H. B. Harris P. Xu M. Z. Charlton M. (2018). Shp2graph: tools to convert a spatial network into an igraph graph in R. ISPRS Int. J. Geo Inf. 7:293. doi: 10.3390/ijgi7080293

[ref50] Magoc T. Salzberg S. L. (2011). FLASH: fast length adjustment of short reads to improve genome assemblies. Bioinformatics 27, 2957–2963. doi: 10.1093/bioinformatics/btr507, 21903629 PMC3198573

[ref51] Martínez-Pérez C. Mohr W. Schwedt A. Dürschlag J. Callbeck C. M. Schunck H. . (2018). Metabolic versatility of a novel N_2_-fixing Alphaproteobacterium isolated from a marine oxygen minimum zone. Environ. Microbiol. 20, 755–768. doi: 10.1111/1462-2920.14008, 29194930

[ref52] Morando M. Magasin J. Cheung S. Mills M. M. Zehr J. P. Turk-Kubo K. A. (2024). Global biogeography of N_2_-fixing microbes: *nifH* amplicon database and analytics workflow. bioRxiv 22:592440. doi: 10.1101/2024.05.04.592440

[ref53] Nielsen L. B. Finster K. Welsh D. T. Donelly A. Herbert R. A. de Wit R. . (2001). Sulphate reduction and nitrogen fixation rates associated with roots, rhizomes and sediments from *Zostera noltii* and *Spartina maritima* meadows. Environ. Microbiol. 3, 63–71. doi: 10.1046/j.1462-2920.2001.00160.x, 11225724

[ref54] Parks D. H. Imelfort M. Skennerton C. T. Hugenholtz P. Tyson G. W. (2015). CheckM: assessing the quality of microbial genomes recovered from isolates, single cells, and metagenomes. Genome Res. 25, 1043–1055. doi: 10.1101/gr.186072.114, 25977477 PMC4484387

[ref55] Postgate J. (1998). The origins of the unit of nitrogen fixation at the University of Sussex. Notes Rec. R. Soc. Lond. 52, 355–362. doi: 10.1098/rsnr.1998.0055

[ref56] R Core Team (2019). R: a language and environment for statistical computing. Vienna: R foundation for statistical computing.

[ref57] Rabalais N. N. Díaz R. J. Levin L. A. Turner R. E. Gilbert D. Zhang J. (2010). Dynamics and distribution of natural and human-caused hypoxia. Biogeosciences 7, 585–619. doi: 10.5194/bg-7-585-2010

[ref58] Reis M. A. M. Almeida J. S. Lemos P. C. Carrondo M. J. T. (1992). Effect of hydrogen-sulfide on growth of sulfate reducing bacteria. Biotechnol. Bioeng. 40, 593–600. doi: 10.1002/bit.260400506, 18601155

[ref59] Samuel T. N. A. Rhys J. P. N. Jakob N. N. Antonio P. C. Gene W. T. Ben J. W. (2024). CoverM: read alignment statistics for metagenomics. Bioinformatics 4:4. doi: 10.1093/bioinformatics/btaf147 PMC11993303 40193404

[ref60] Sarma V. V. S. S. Rao G. D. Viswanadham R. Sherin C. K. Salisbury J. Omand M. M. . (2016). Effects of freshwater stratification on nutrients, dissolved oxygen, and phytoplankton in the bay of Bengal. Oceanography 29, 222–231. doi: 10.5670/oceanog.2016.54

[ref61] Saxena H. Sahoo D. Nazirahmed S. Chaudhari D. Rahi P. Kumar S. . (2023). The bay of Bengal: an enigmatic diazotrophic niche. J. Geophys. Res. Biogeosci. 128:7687. doi: 10.1029/2023JG007687

[ref62] Schlitzer R. (2018). Ocean data view [software]. ODV

[ref63] Segata N. Izard J. Waldron L. Gevers D. Miropolsky L. Garrett W. S. . (2011). Metagenomic biomarker discovery and explanation. Genome Biol. 12:R60. doi: 10.1186/gb-2011-12-6-r60, 21702898 PMC3218848

[ref64] Severin I. Confurius-Guns V. Stal L. J. (2012). Effect of salinity on nitrogenase activity and composition of the active diazotrophic community in intertidal microbial mats. Arch. Microbiol. 194, 483–491. doi: 10.1007/s00203-011-0787-5, 22228487 PMC3354318

[ref65] Shi D. L. Hu X. H. Wen Z. Z. Hong H. Z. (2021). Marine biological nitrogen fixation and its response to global change. J. Xiamen Univ. 60, 367–381. doi: 10.6043/j.issn.0438-0479.202010015

[ref66] Shiau Y. J. Lin M. F. Tan C. C. Tian G. L. Chiu C. Y. (2017). Assessing N_2_ fixation in estuarine mangrove soils. Estuar. Coast. Shelf Sci. 189, 84–89. doi: 10.1016/j.ecss.2017.03.005

[ref67] Slobodkin A. I. Reysenbach A. L. Slobodkina G. B. Kolganova T. V. Kostrikina N. A. Bonch-Osmolovskaya E. A. (2013). *Dissulfuribacter thermophilus* gen. Nov., sp nov., a thermophilic, autotrophic, sulfur-disproportionating, deeply branching deltaproteobacterium from a deep-sea hydrothermal vent. Int. J. Syst. Evol. Microbiol. 63, 1967–1971. doi: 10.1099/ijs.0.046938-0, 23024145

[ref68] Song T. Liang Q. Du Z. Wang X. Chen G. Du Z. . (2022). Salinity gradient controls microbial community structure and assembly in coastal solar Salterns. Genes 13:385. doi: 10.3390/genes13020385, 35205428 PMC8872224

[ref69] Staal M. Meysman F. J. R. Stal L. J. (2003). Temperature excludes N_2_-fixing heterocystous cyanobacteria in the tropical oceans. Nature 425, 504–507. doi: 10.1038/nature01999, 14523445

[ref70] Stramma L. Johnson G. C. Sprintall J. Mohrholz V. (2008). Expanding oxygen-minimum zones in the tropical oceans. Science 320, 655–658. doi: 10.1126/science.1153847, 18451300

[ref71] Subramaniam A. Yager P. L. Carpenter E. J. Mahaffey C. Björkman K. Cooley S. . (2008). Amazon River enhances diazotrophy and carbon sequestration in the tropical North Atlantic Ocean. Proc. Natl. Acad. Sci. USA 105, 10460–10465. doi: 10.1073/pnas.0710279105, 18647838 PMC2480616

[ref72] Sun Y. H. Du P. Li H. L. Zhou K. L. Shou L. Chen J. F. . (2024). Prokaryotic community assembly patterns and nitrogen metabolic potential in oxygen minimum zone of Yangtze estuary water column. Environ. Res. 252:119011. doi: 10.1016/j.envres.2024.119011 38670213

[ref73] Sun Z. Zhu Y. Jiang Y. Zhai H. Chen J. Yan X. . (2025). Intrusion of Kuroshio enhances phytoplankton biomass and diversity in the East China Sea. J. Geophys. Res. Oceans 130:1337. doi: 10.1029/2024JC021337, 40890438

[ref74] Thompson P. L. Guzman M. Meester L. D. Zsófia Horváth Chase J. M. (2020). A process-based metacommunity framework linking local and regional scale community ecology. Cold Spring Harbor Laboratory 9:2170. doi: 10.1101/832170 PMC7496463 32672410

[ref75] Uritskiy G. V. DiRuggiero J. Taylor J. (2018). MetaWRAP-a flexible pipeline for genome-resolved metagenomic data analysis. Microbiome 6:158. doi: 10.1186/s40168-018-0541-1, 30219103 PMC6138922

[ref76] Villanueva R. A. M. Chen Z. J. (2019). Ggplot2: elegant graphics for data analysis, 2nd edition. Measurement Interdisciplinary Res. Persp. 17, 160–167. doi: 10.1080/15366367.2019.1565254

[ref77] Voss M. Bombar D. Loick N. Dippner J. W. (2006). Riverine influence on nitrogen fixation in the upwelling region off Vietnam, South China Sea. Geophys. Res. Lett. 33:5569. doi: 10.1029/2005gl025569

[ref78] Waite D. W. Chuvochina M. Pelikan C. Parks D. H. Yilmaz P. Wagner M. . (2020). Proposal to reclassify the proteobacterial classes Deltaproteobacteria and Oligoflexia, and the phylum Thermodesulfobacteria into four phyla reflecting major functional capabilities. Int. J. Syst. Evol. Microbiol. 70, 5972–6016. doi: 10.1099/ijsem.0.004213, 33151140

[ref79] Wang S. S. Jiang L. J. Zhao Z. M. Chen Z. Wang J. Alain K. . (2024). Chemolithoautotrophic diazotrophs dominate dark nitrogen fixation in mangrove sediments. ISME J. 18:119. doi: 10.1093/ismejo/wrae119, 38916247 PMC11474244

[ref80] Wu C. Kan J. J. Liu H. J. Pujari L. Guo C. C. Wang X. Z. . (2019). Heterotrophic bacteria dominate the diazotrophic community in the eastern Indian Ocean (EIO) during pre-southwest monsoon. Microb. Ecol. 78, 804–819. doi: 10.1007/s00248-019-01355-1, 31037377

[ref81] Wu D. Wang Y. G. Shao Y. Wang Y. Hu G. (2022). Simulation and statistical analysis on the transport process of salt water mass from the north branch in the Yangtze River estuary. J. Phys. Conf. Ser. 2224:2065. doi: 10.1088/1742-6596/2224/1/012065

[ref82] Xie C. Ouyang H. Zheng H. Wang M. Gu J. Wang Z. . (2023). Community structure and association network of prokaryotic community in surface sediments from the Bering-Chukchi shelf and adjacent sea areas. Front. Microbiol. 14:1312419. doi: 10.3389/fmicb.2023.1312419, 38264483 PMC10803617

[ref83] Xue C. X. Lin H. Y. Zhu X. Y. Liu J. W. Zhang Y. H. Rowley G. . (2021). Diting: a pipeline to infer and compare biogeochemical pathways from metagenomic and metatranscriptomic data. Front. Microbiol. 12:698286. doi: 10.3389/fmicb.2021.698286, 34408730 PMC8367434

[ref84] Yang Q. S. Dong J. D. Ahmad M. Ling J. Zhou W. G. Tan Y. H. . (2019). Analysis of *nifH* DNA and RNA reveals a disproportionate contribution to nitrogenase activities by rare plankton-associated diazotrophs. BMC Microbiol. 19:188. doi: 10.1186/s12866-019-1565-9, 31416417 PMC6694519

[ref85] Zehr J. P. Capone D. G. (2020). Changing perspectives in marine nitrogen fixation. Science 368:729. doi: 10.1126/science.aay9514 32409447

[ref86] Zehr J. P. Capone D. G. (2024). Unsolved mysteries in marine nitrogen fixation. Trends Microbiol. 32, 532–545. doi: 10.1016/j.tim.2023.08.004, 37658011

[ref87] Zehr J. P. Mcreynolds L. A. (1989). Use of degenerate oligonucleotides for amplification of the *nifH* gene from the marine cyanobacterium *Trichodesmium thiebautii*. Appl. Environ. Microbiol. 55, 2522–2526. doi: 10.1128/Aem.55.10.2522-2526.1989, 2513774 PMC203115

[ref88] Zehr J. P. Turner P. J. (2001). Nitrogen fixation: Nitrogenase genes and gene expression. Methods Microbiol. 30, 271–286. doi: 10.1016/S0580-9517(01)30049-1

[ref89] Zhang J. Gilbert D. Gooday A. J. Levin L. Naqvi S. W. A. Middelburg J. J. . (2010). Natural and human-induced hypoxia and consequences for coastal areas: synthesis and future development. Biogeosciences 7, 1443–1467. doi: 10.5194/bg-7-1443-2010

[ref90] Zhao R. Jrgensen S. L. Babbin A. R. (2024). An abundant bacterial phylum with nitrite-oxidizing potential in oligotrophic marine sediments. Commun. Biol. 7:6136. doi: 10.1038/s42003-024-06136-2 PMC11009272 38605091

[ref91] Zhou Y. T. Gong H. J. Zhou F. (2022). Responses of horizontally expanding oceanic oxygen minimum zones to climate change based on observations. Geophys. Res. Lett. 49:7724. doi: 10.1029/2022GL097724

[ref92] Zhou Z. C. Tran P. Q. Liu Y. Kieft K. Anantharaman K. (2019). Metabolic: a scalable high-throughput metabolic and biogeochemical functional trait profiler based on microbial genomes. bioRxiv 22:761643. doi: 10.1101/761643

