# Correction: Chemoautotrophic Thermodesulfobacteriota as a key genomic potential group in the hypoxic diazotrophic community of the Changjiang (Yangtze River) estuary

**DOI:** 10.3389/fmicb.2025.1766907

**Published:** 2026-01-12

**Authors:** Mengjia Zhang, Yuanli Zhu, Zhenhao Sun, Bin Wang, Jianfang Chen, Feng Zhou, Jiangning Zeng, Meng Li, Dayu Zou, Zhibing Jiang

**Affiliations:** 1 Key Laboratory of Marine Ecosystem Dynamics, Second Institute of Oceanography, Ministry of Natural Resources, Hangzhou, China; 2 Key Laboratory of Nearshore Engineering Environment and Ecological Security of Zhejiang Province, Hangzhou, China; 3 State Key Laboratory of Satellite Ocean Environment Dynamics, Second Institute of Oceanography, Ministry of Natural Resources, Hangzhou, China; 4 Observation and Research Station of Marine Ecosystem in the Yangtze River Delta, Ministry of Natural Resources, Hangzhou, China; 5 Archaeal Biology Centre, Synthetic Biology Research Center, Shenzhen Key Laboratory of Marine Microbiome Engineering, Key Laboratory of Marine Microbiome Engineering of Guangdong Higher Education Institutes, Institute for Advanced Study, Shenzhen University, Shenzhen, China; 6 Key Laboratory of Ocean Space Resource Management Technology, Ministry of Natural Resource, Marine Academy of Zhejiang Province, Hangzhou, China

**Keywords:** coastal hypoxia, nitrogen fixation, diazotrophic biodiversity, Thermodesulfobacteriota, metabarcoding and metagenomic analysis

Zhibing Jiang was erroneously assigned to affiliation five, “Archaeal Biology Centre, Synthetic Biology Research Center, Shenzhen Key Laboratory of Marine Microbiome Engineering, Key Laboratory of Marine Microbiome Engineering of Guangdong Higher Education Institutes, Institute for Advanced Study, Shenzhen University, Shenzhen, China”.

Zhibing Jiang's Affiliation “Key Laboratory of Ocean Space Resource Management Technology, Ministry of Natural Resource, Marine Academy of Zhejiang Province, Hangzhou, China” was omitted.

The original version of this article has been updated.

