# Supplementary material for: Chemoautotrophic Thermodesulfobacteriota as a key genomic potential group in the hypoxic diazotrophic community of the Changjiang (Yangtze River) estuary
Source: Front Microbiol. 2025 Dec 4;16:1671267. doi: 10.3389/fmicb.2025.1671267 (PMC12711143; doi:10.3389/fmicb.2025.1671267)
Supplement: Supplementary file 1 [file Supplementary_file_1.docx]

Supplementary Material

# Supplementary Figures and Tables

## Supplementary Figures


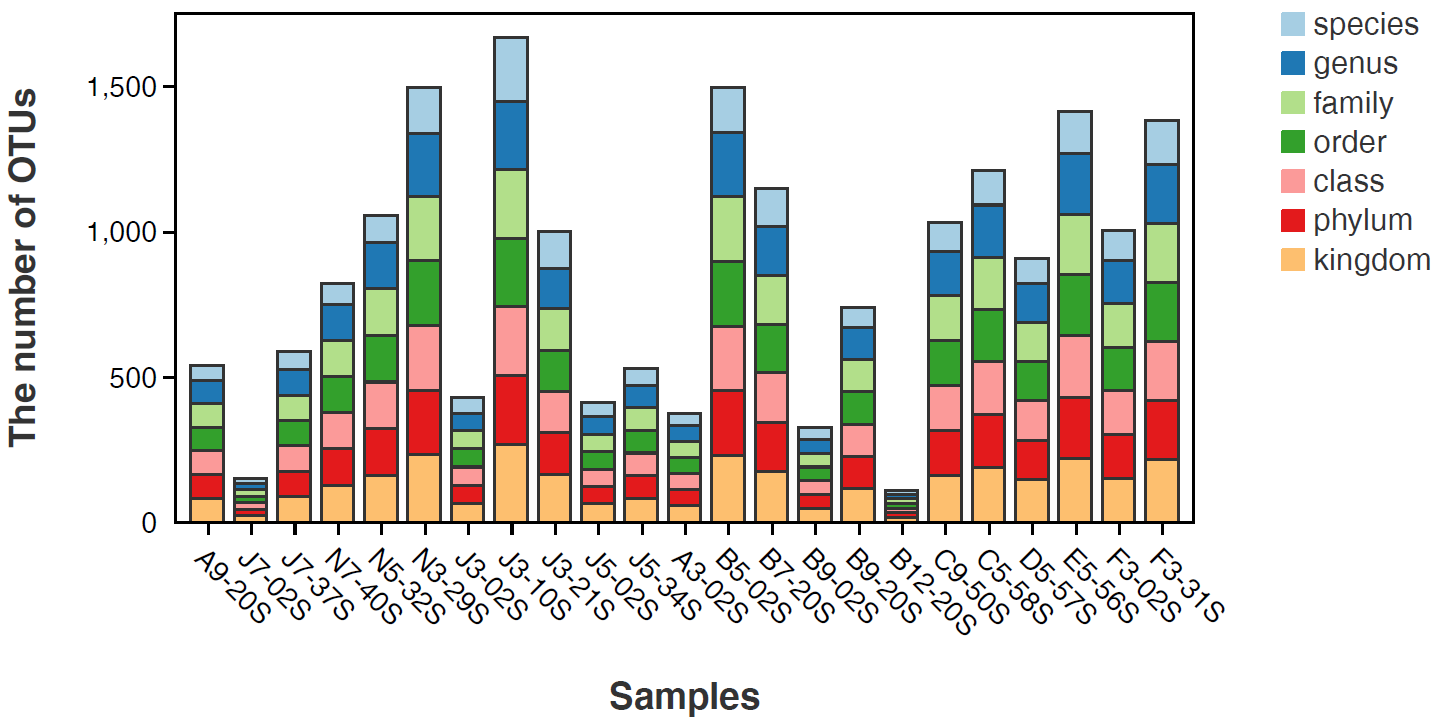


**Supplementary Figure 1.** Taxonomic resolution of diazotrophic communities across classification levels. The majority of sequences (93.0%) achieved genus-level classification, with resolution decreasing at finer scales (species: 71.6%). Histograms depict the percentage of high-quality *nifH* sequences successfully annotated at each taxonomic rank (phylum to genus) for 23 water samples; Colors represent different taxa; Taxonomic assignments were derived from BLASTn against NCBI NT and *nifH* ASV databases (e-value = 1e-5, qcov = 95%, identity = 99%).


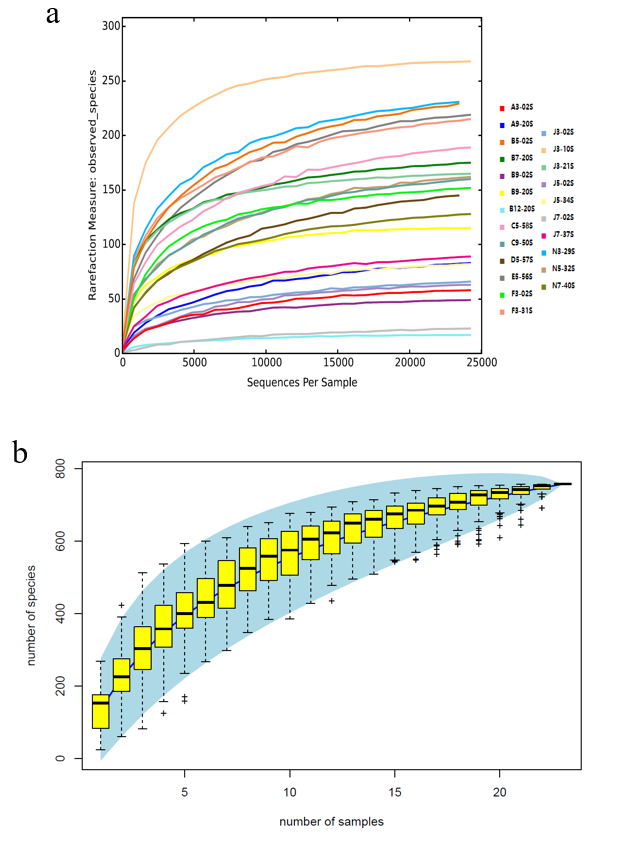


**Supplementary Figure 2.** Sampling sufficiency and sequencing depth validation for diazotrophic communities. (a) Rarefaction curves demonstrate asymptotic species richness accumulation at ~10,000 sequences per sample, indicating adequate sequencing depth. Different colors of lines represent different samples; (b) Species accumulation curves plateau at 23 samples, confirming comprehensive community capture. Blue bands represent 95% confidence intervals; Analyses was performed using the VEGAN package (Dixon, 2003) with 1,000 permutations.


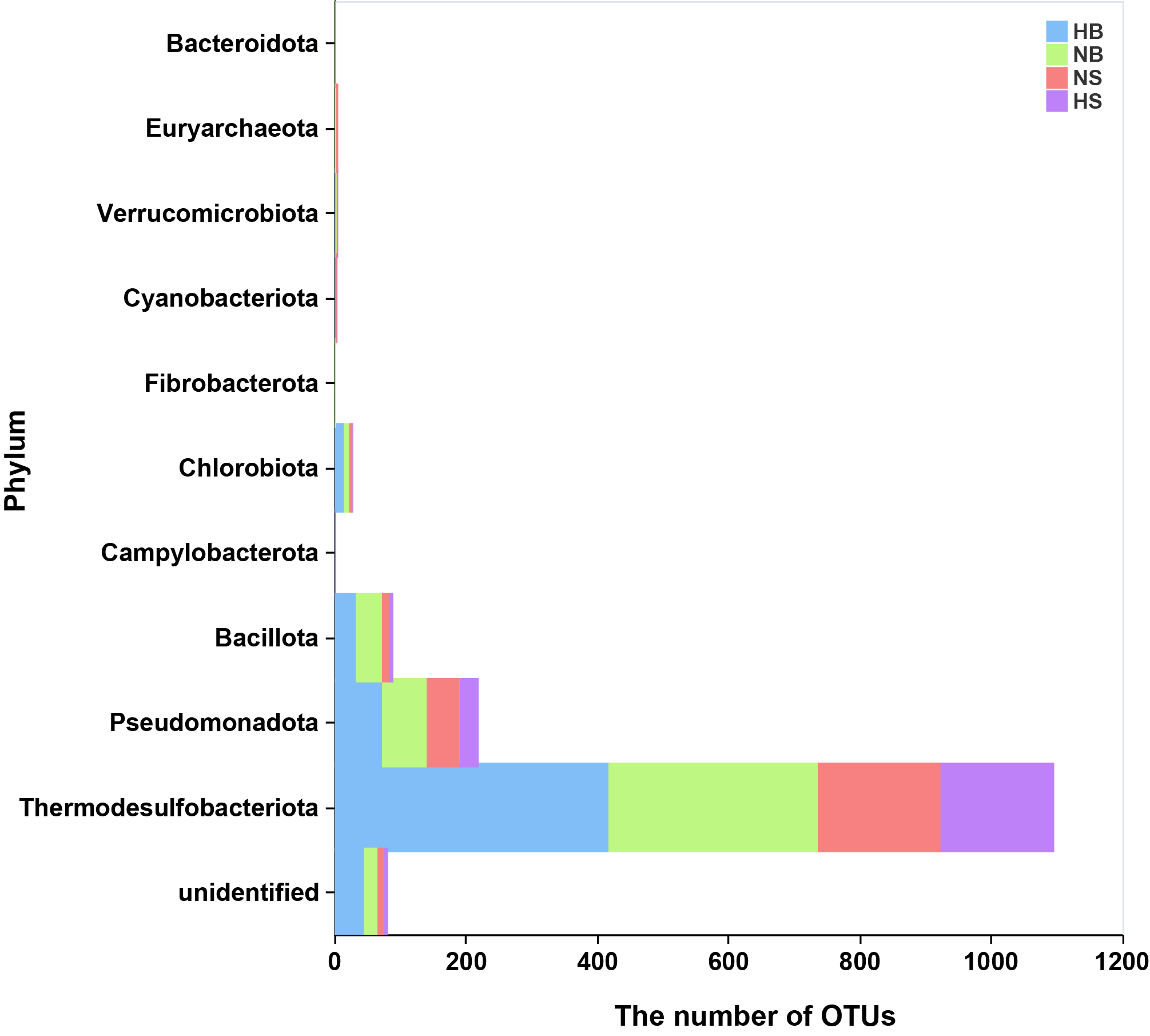


**Supplementary Figure 3.** Richness of different phylum in the different areas in Changjiang Estuary. Bar heights represent taxonomic richness (number of OTUs). HB = Hypoxic-Bottom, represents hypoxic zone (all in the bottom, DO < 62.5 μmol L⁻¹); HS = Hypoxic-Surface, represents surface layers above the hypoxic zone without hypoxia; NB = Non-hypoxic-Bottom, represents bottom layers in the non-hypoxic zone; NS = Non-hypoxic-Surface, represents surface layers in the non-hypoxic zone.


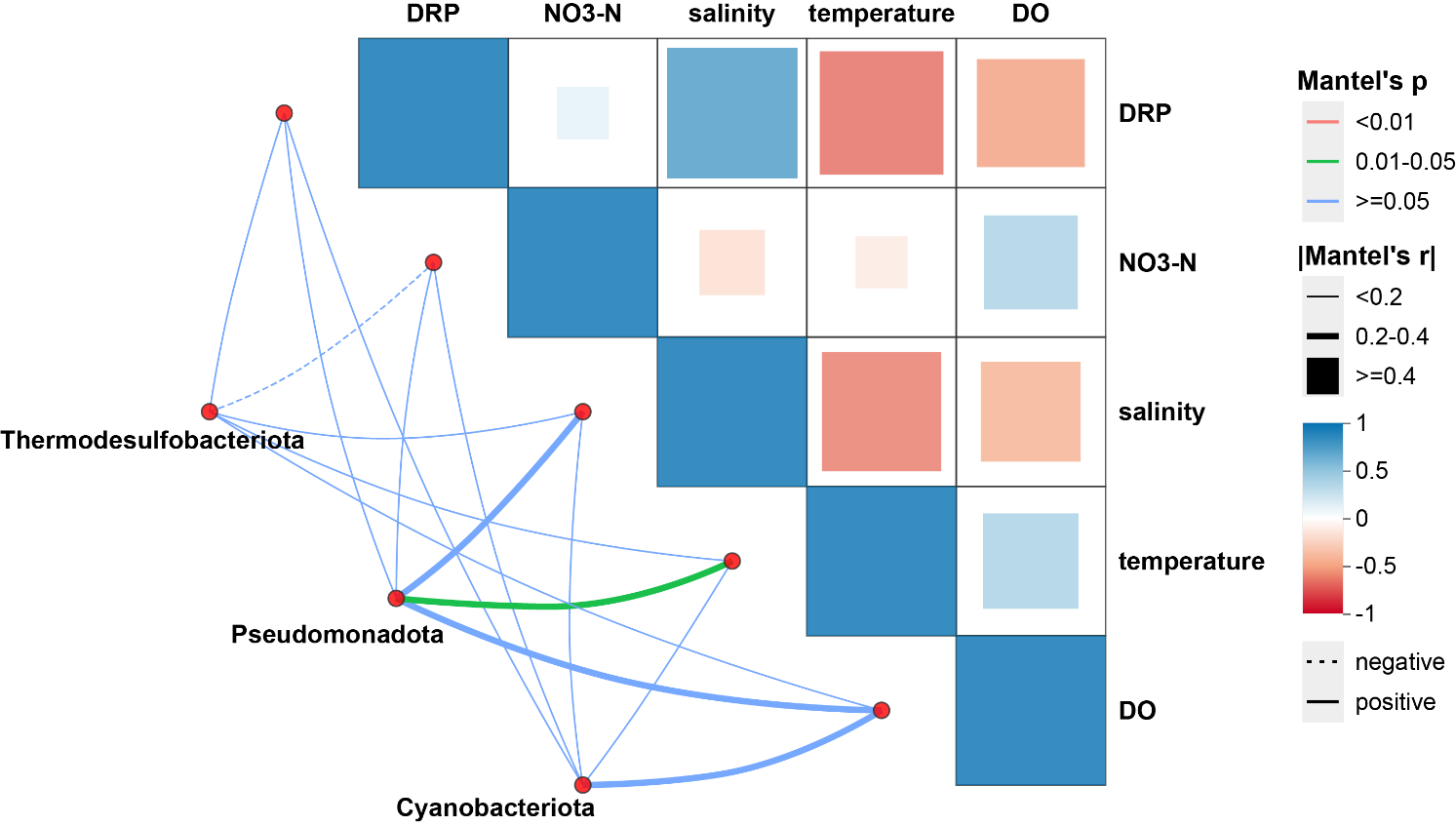


**Supplementary Figure 4.** Correlation between environmental factors and diazotrophic communities. Mantel test on interactions of three phyla (Thermodesulfobacteriota, Pseudomonadota, and Cyanobacteriota) with environmental factors. The color of the lines represents the p-value, the thickness represents the r-value, and the style (solid versus dashed) denotes positive versus negative correlations, respectively.

**
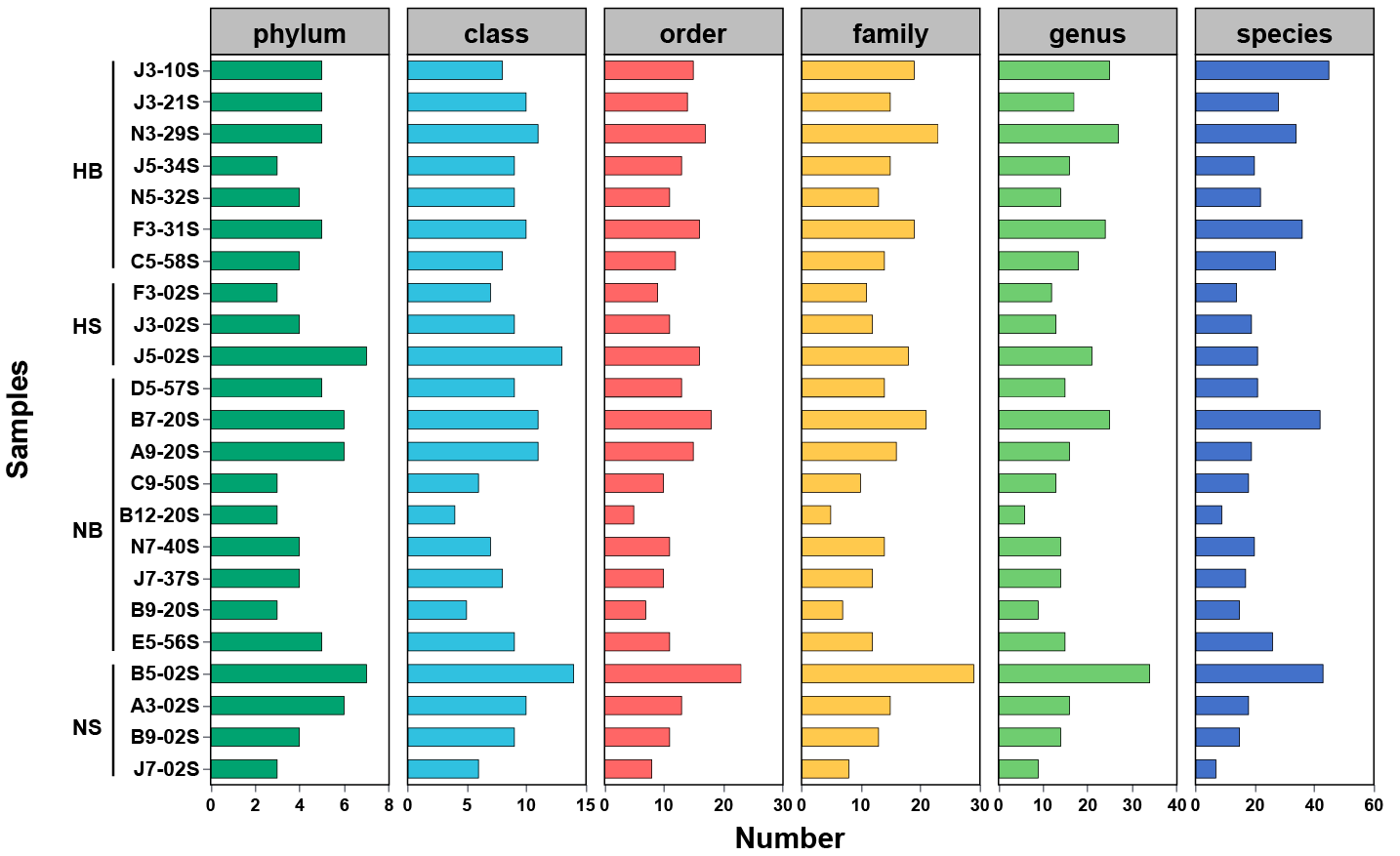
**

**Supplementary Figure 5.** The number of different classification levels in different samples. As the taxonomic ranks become more and more refined, the quantitative differences between different samples become greater. Colors represent different taxonomic ranks; HB = Hypoxic-Bottom, represents hypoxic zone (all in the bottom, DO < 62.5 μmol L⁻¹); HS = Hypoxic-Surface, represents surface layers above the hypoxic zone without hypoxia; NB = Non-hypoxic-Bottom, represents bottom layers in the non-hypoxic zone; NS = Non-hypoxic-Surface, represents surface layers in the non-hypoxic zone.


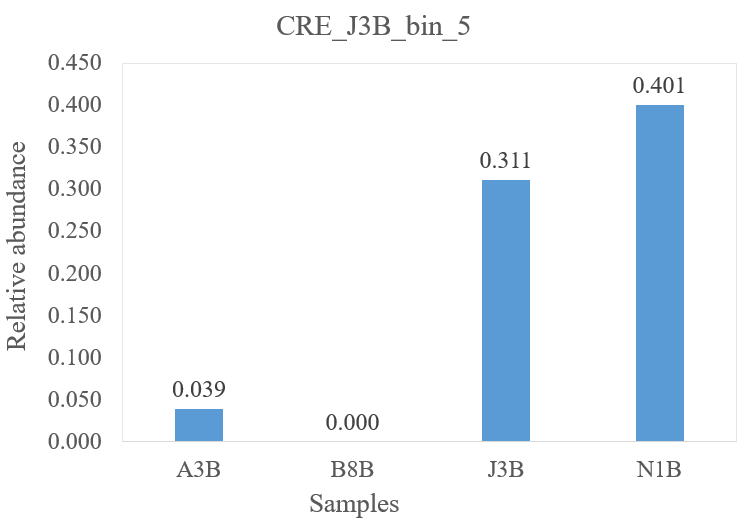


**Supplementary Figure 6.** Hypoxia-driven enrichment of a Thermodesulfobacteriota metagenome-assembled genome (MAG, CRE_J3B_bin_5) with *nifH* gene. Bar plot compares the relative abundance of MAG CRE_J3B_bin_5 (classified to Desulfocapsaceae, Thermodesulfobacteriota) across hypoxic (J3 and N1) and non-hypoxic (A3 and B8) samples. This MAG exhibited 18.25-fold higher relative abundance in hypoxic zone compared to non-hypoxic zone, indicating strong niche-specific adaptation and proliferation under low-oxygen conditions. Relative abundance was calculated as RPKM (Reads Per Kilobase per Million mapped reads) values using CoverM (Samuel et al., 2024).

## Supplementary Tables

**Supplementary Table 1.** Adonis analysis results for all environmental factors

|  | Df | SumOfSqs | R^2^ | F | Pr(>F) |
| --- | --- | --- | --- | --- | --- |
| DO | 1 | 0.7271 | 0.0969 | 2.5887 | 0.001 |
| Temperature | 1 | 0.6332 | 0.08439 | 2.2544 | 0.001 |
| Salinity | 1 | 0.4818 | 0.06422 | 1.7155 | 0.012 |
| NO3^−^ | 1 | 0.5034 | 0.06709 | 1.7923 | 0.013 |
| DRP | 1 | 0.383 | 0.05105 | 1.3638 | 0.1 |
| Residual | 17 | 4.7747 | 0.63635 |  |  |
| Total | 22 | 7.5032 | 1 |  |  |

Note: Df = degrees of freedom; SumOfSqs = total variance; R^2^ = variance contribution, indicating the extent to which different groups explain the differences among samples; F = F-test value; Pr (>F) = significance p-value, *P* < 0.05 indicates a significant difference.

**Supplementary Table 2.** Representative metagenome assembled genomes (MAGs) annotated to Desulfobacterota (Thermodesulfobacteriota in NCBI)

| user_genome | classification | msa_  percent | *nifH* |
| --- | --- | --- | --- |
| CRE_A3B_bin_51 | d__Bacteria;p__Desulfobacterota_B;c__Binatia;o__UBA12015;f__UBA12015;g__VGTG01;s__ | 93.58 | Absent |
| CRE_A3B_bin_69 | d__Bacteria;p__Desulfobacterota_B;c__Binatia;o__UBA12015;f__UBA12015;g__UBA12015;s__ | 64.63 | Absent |
| CRE_A3B_bin_98 | d__Bacteria;p__Desulfobacterota_B;c__Binatia;o__UBA12015;f__UBA12015;g__VGTG01;s__ | 92.29 | Absent |
| CRE_J3B_bin_112 | d__Bacteria;p__Desulfobacterota;c__Desulfobulbia;o__Desulfobulbales;f__Desulfocapsaceae;g__Desulforhopalus;s__ | 88.6 | Absent |
| CRE_J3B_bin_189 | d__Bacteria;p__Desulfobacterota;c__Desulfobulbia;o__Desulfobulbales;f__Desulfocapsaceae;g__Desulforhopalus;s__ | 48.18 | Absent |
| **CRE_J3B_bin_5** | **d__Bacteria;p__Desulfobacterota;c__Desulfobulbia;o__Desulfobulbales;f__Desulfocapsaceae;g__Desulforhopalus;s__** | **86.89** | **Present** |
| CRE_J3B_bin_70 | d__Bacteria;p__Desulfobacterota;c__Desulfobacteria;o__Desulfobacterales;f__Desulfobacteraceae;g__Desulfobacula;s__ | 75.55 | Absent |
| CRE_J3B_bin_9 | d__Bacteria;p__Desulfobacterota;c__Desulfobulbia;o__Desulfobulbales;f__Desulfocapsaceae;g__Desulfotalea;s__ | 85.14 | Absent |
| CRE_N1B_bin_116 | d__Bacteria;p__Desulfobacterota_B;c__Binatia;o__UBA1149;f__SPBQ01;g__SPBQ01;s__ | 59.64 | Absent |
| CRE_N1B_bin_30 | d__Bacteria;p__Desulfobacterota_B;c__Binatia;o__UBA12015;f__UBA12015;g__VGTG01;s__VGTG01 sp029976905 | 88.38 | Absent |
| CRE_N1B_bin_60 | d__Bacteria;p__Desulfobacterota;c__Desulfobulbia;o__Desulfobulbales;f__Desulfocapsaceae;g__Desulfopila;s__ | 51.04 | Absent |

**Supplementary Table 3.** Contribution of Desulfocapsaceae (family) to different metabolic processes (Top 20)

| Function | MW-score of Desulfocapsaceae |
| --- | --- |
| C-S-02:Carbon fixation - Wood-Ljungdahl pathway | 57.6 |
| C-S-02:Carbon fixation - 3HP/4HB | 36.8 |
| **N-S-01:N2 fixation - nifDK\|\|vnfDKG\|\|nifH** | **34.7** |
| S-S-06:Sulfite reduction - dsrABD | 25.9 |
| O-S-04:Arsenite oxidation | 11 |
| O-S-03:Arsenate reduction | 9 |
| C-S-09:Hydrogen oxidation | 4.4 |
| O-S-05:Selenate reduction | 4.1 |
| S-S-08:Thiosulfate disproportionation (to sulfite + hydrogen sulfide) | 2.7 |
| S-S-04:Sulfite oxidation | 2.5 |
| S-S-05:Sulfate reduction | 2.5 |
| C-S-01:Organic carbon oxidation - aromatics degradation | 2 |
| N-S-04:Nitrate reduction - napAB | 2 |
| S-S-01:Sulfide oxidation - sqr | 1.8 |
| C-S-03:Ethanol oxidation | 1.6 |
| O-S-01:Iron reduction | 1.5 |
| C-S-01:Organic carbon oxidation - formate oxidation | 1.4 |
| O-S-02:Iron oxidation: | 1.4 |
| N-S-05:Nitrite reduction - octR | 1.3 |
| S-S-03:Sulfur oxidation - sdo | 1.3 |

Note: MW-score = metabolic weight score, serving as metrics to measure function weights.
